# Diversity and functional potential of bacterial and fungal endophytes in traditional food wrapping leaves reveal implications for artisanal food safety and quality

**DOI:** 10.3389/fmicb.2026.1641069

**Published:** 2026-04-29

**Authors:** Rasheed A. Adeleke, Thabang M. E. Machailoe, Michelle Malemagovha, Oluwaseyi S. Olanrewaju, Kazeem A. Alayande, Linda U. Obi, Oluwadamilola M. Makinde

**Affiliations:** Unit for Environmental Sciences and Management, North-West University (Potchefstroom Campus), Potchefstroom, South Africa

**Keywords:** bacteria, endophytes, food safety, fungi, ready-to-eat foods, wrapping leaves

## Abstract

Plant leaves are widely utilised globally for the packaging and serving of traditionally prepared foods. The microbial communities associated with these wrapping leaves, particularly endophytes, are recognised to potentially influence food quality, safety, and preservation. Specifically, certain endophytes can enhance sensory attributes and nutritional value through fermentative processes, while the presence of harmful microorganisms may lead to spoilage and pose a risk of foodborne illness. This study utilised 16S rRNA, ITS metabarcoding and metagenomic functional analysis (PICRUSt2) to comprehensively investigate the composition and infer the putative functional potential of putative endophytic bacterial and fungal communities present in 53 samples of four different food wrapping leaves. The leaves examined included *Thaumatococcus daniellii* (*n* = 10), *Alstonia macrophylla* (*n* = 18), *Theobroma species* (*n* = 14), and *Megaphrynium macrostachyum* (*n* = 11). Distinct microbial community profiles were observed across the different leaf types. Highest bacterial species richness and community variability were detected in *A. macrophylla* samples, reflected by Principal Coordinates Analysis (PCoA) values (PCoA1 = 43.97%; PCoA2 = 10.68%). Conversely, *M. macrostachyum* exhibited the greatest fungal species richness and variability (PCoA1 = 20.08%; PCoA2 = 8.72%). Taxonomic analysis identified Proteobacteria as the dominant bacterial phylum and *Stenotrophomonas* as the dominant bacterial genus. Other notable bacterial taxa included the phyla Bacteroidota and Firmicutes, and genera such as *Pseudomonas, Faecalibacterium*, and *Bacteroides*. For fungal communities, Ascomycota was the dominant phylum. Additional fungal taxa included the phylum Basidiomycota and genera Cryptococcus, Candida, and Meyerozyma. A core microbiome analysis revealed that 42 bacterial (notably Stenotrophomonas and Chryseobacterium) and 7 fungal taxa (notably Pleosporaceae and Ascomycota) were shared across all examined wrapping leaves. The identified microbial communities (e.g., *Lactobacillus* and *Geotrichum*) encompass taxa with potential beneficial roles, such as enhancing food fermentation and potentially contributing to human gut health upon consumption of the packaged food. However, the detection of potentially pathogenic and toxigenic bacterial taxa highlights a possible public health risk associated with the use of these leaves. Further investigation into the specific functionalities of these associated bacteria and fungi is essential to maximise their beneficial applications while simultaneously mitigating potential health risks posed by harmful strains.

## Introduction

Traditional or artisanal-produced foods, characterised by small-scale, local processing with an emphasis on tradition and authenticity, are significant globally (Lindbergh and Schwartz, 2021). Food packaging plays a critical role in defining the dietary appeal, safety, and shelf-life of traditional food products, with wrapping leaves employed for centuries in enhancing organoleptic quality and aiding preservation (Ogunlade and Adeleke, 2021; Saha, 2022).

The practise of using leaves for wrapping and serving traditional foods is widespread, with typical examples including the use of miracle berry leaves (*Thaumatococcus daniellii*) and matchstick leaves (*Megaphrynium macrostachyum*) for packaging eko and aadun in Nigeria, banana leaves (*Musa paradisiaca*) for *Parkia biglobosa* seeds (iru) and Kenkey in Nigeria and Ghana, respectively, banana/plantain leaves (*Musa* species) for Dangke cheese in Indonesia, and Indian shot leaves (*Canna indica*) for Tamales in Mexico (Hounsou et al., 2022; Lascurain et al., 2017; Makinde et al., 2023; Mensah et al., 2012; Ogunlade et al., 2023). Beyond their biodegradability and low chemical toxicity, a crucial, yet often overlooked, aspect influencing the safety and quality of food wrapped in leaves is their microbial composition, particularly the presence of endophytes (Septiana et al., 2017; Strobel and Daisy, 2003). Despite artisanal food processing steps (e.g., steaming and fermentation) selectively eliminating heat and low pH-sensitive endophytes, respectively, spore-forming and acid-tolerant species from the wrapping leaves may complement the dominant fermenting organisms or compete with spoilage organisms (Zhou et al., 2024).

Endophytes (Greek: endon = within; phyton = plant) are microorganisms, including fungi and bacteria, that establish endosymbiotic relationships with plants by colonising and residing within plant tissues, either transiently or persistently, during their life cycle without causing obvious disease symptoms (Mercado-Blanco, 2014; Card et al., 2016; Agrawal and Bhatt, 2023). These endosymbiotic relationships are typically mutualistic, in which endophytes are more likely to benefit the host plant than to cause harm. Plant-endophyte interactions are multifaceted and can be obligate or facultative, with diverse endophytic taxa exhibiting organ-specific associations within the plant, including roots, stems, flowers, fruits, seeds, and leaves (Agrawal and Bhatt, 2023; Mensah et al., 2012; Mushtaq et al., 2023).

On wrapping leaves, endophytes can influence the packaged food directly through the production of metabolites that may be transferred into the food. A notable example is thaumatin, a sweetener protein associated with the fruit of *Thaumatococcus daniellii*, however, leaf associated fungi such as *Penicillium roqueforti* and *Aspergillus luchuensis* (formerly *A. awamori*) known to produce thaumatin-like proteins (Faus et al., 1998; Faus, 2000; Yamada et al., 2016; Sanchez and Demain, 2017). Leaves, such as those from *Thaumatococcus daniellii*, have also been reported to extend the shelf life of food more effectively than some synthetic packaging materials (Yusli et al., 2023; Famuwagun et al., 2024). This potential for shelf-life extension is evident in medicinal plants, where bioactive compounds derived from endophytic fungi have demonstrated antibacterial, antifungal, and antioxidant activities (Kumar and Prasher, 2023). However, the microbial communities on wrapping leaves can also include endophytes that pose potential risks, including foodborne illness from pathogenic species (e.g., *Pseudomonas aeruginosa*) and mycotoxin contamination from toxigenic genera such as *Alternaria alternata*. *Pseudomonas* and *Alternari*a have been identified as potentially pathogenic and toxigenic, respectively, thereby representing a potential health risk to consumers of food wrapped in these leaves (DeMers, 2022; Xiang et al., 2022). While washing leaves before use may affect epiphytic populations on the surface, it is unlikely to significantly affect endophyte populations, as they reside within the plant tissue. During food preparation such as steaming, fermentation, or prolonged food-leaf contact, endophytic microbes can be released into the food matrix. Understanding the diversity of bacterial and fungal endophytes associated with wrapping leaves is crucial as such knowledge will provide critical insight into the influence these microorganisms can have on the quality and safety of artisanal produced food. Ultimately, this understanding contributes to achieving Sustainable Development Goal (SDG) 3, which aims to ensure good health and well-being for all globally. This study therefore aims to determine the diversity and potential functions of bacterial and fungal endophytes associated with selected food wrapping leaves, as existing studies lack such data.

## Materials and methods

### Sample collection and preparation

**Figure 1:**
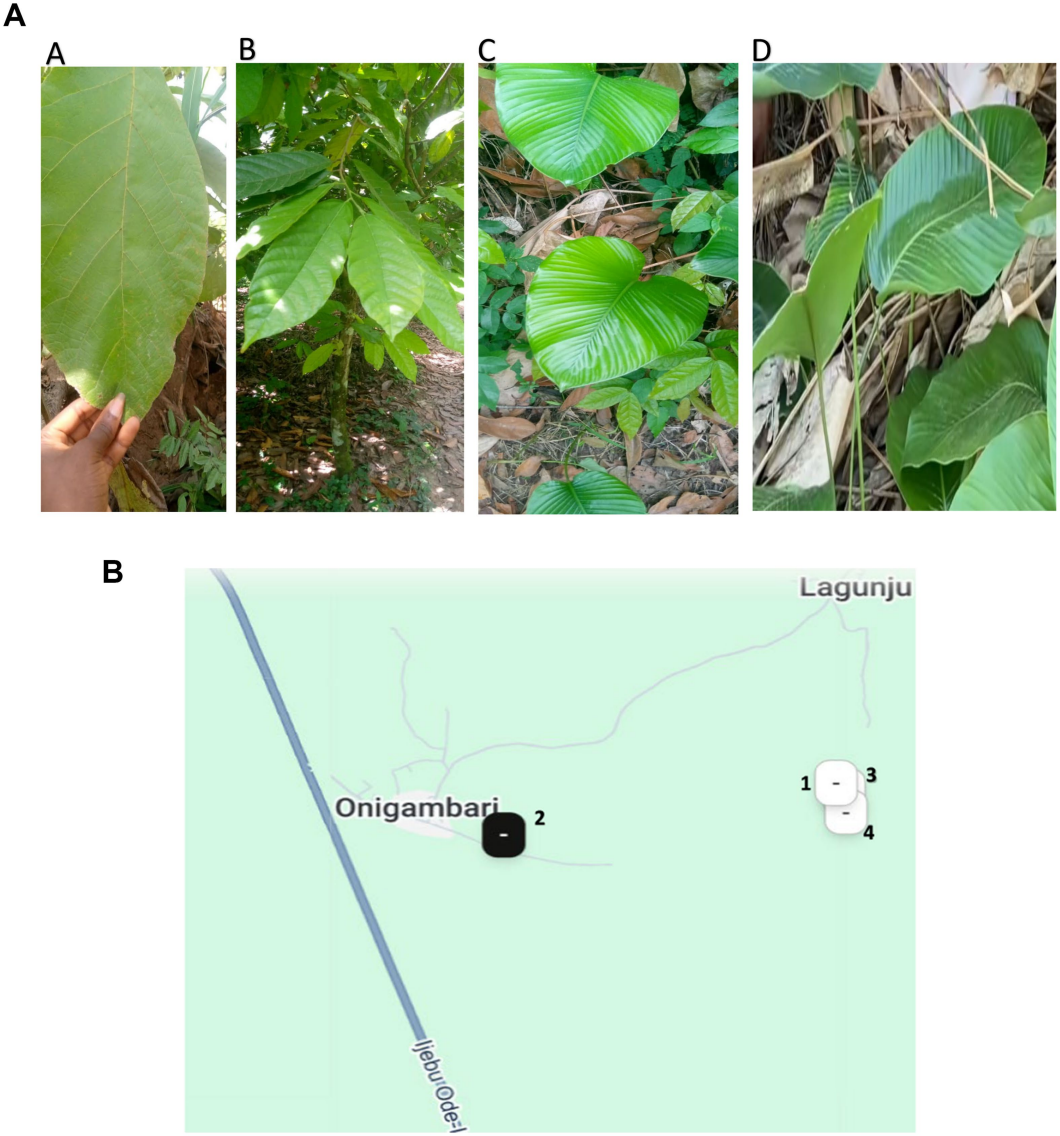
**(A)** A: *Alstonia macrophylla* (*Am*). B: *Theobroma* species (*Ts*), C: *Megaphrynium macrostachyum* (*Mm*), D: *Thaumatococcus daniellii* (*Td*). **(B)** Map showing the sites were wrapping leaves collection sites. 1: *Thaumatococcus daniellii* (7.1379°N, 3.9110°E). 2: *Alstonia macrophylla* (7.1351°N, 3.8967°E), 3: *Theobroma* species (7.1363°N, 3.9114°E), 4: *Megaphrynium macrostachyum* (7.1374°N, 3.9113°E).

A total of 53 food wrapping leaves comprising *Ewe Eran* (*Thaumatococcus daniellii,* Td) (*n* = 10) 7°08′16.3”N 3°54′39.5″E, Matchstick leave (*Alstonia macrophylla,* Am) (*n* = 18) 7°08′06.5”N 3°53′48.0″E, Cocoa leaves (*Theobroma* species, Ts) (*n* = 14) 7°08′10.7”N 3° 54′41.1″E and *Ewe gbodogi* (*Megaphrynium macrostachyum,* Mm) (*n* = 11) 7°08′14.6”N 3°54′40.7″E were collected from different farms as shown in Figures 1A,B. The leaves were selected by observation of mature, visually healthy plants, with consistent leaf size wide enough for food packaging. The leaves were surface disinfected within a period of 24 h according to the method of Saldierna Guzmán et al. (2020) before transporting them on ice to the microbiology laboratory at the North-West University, Potchefstroom, South Africa. The surface disinfection protocol includes careful washing of the leaves using tap water to remove dust and debris then dried in the air. Samples were then disinfected by immersing them sequentially in 70% ethanol for 3 min, 0.4% NaOCl for 1 min, and 70% ethanol for 2 min. They were then washed three times with sterile distilled water for 1 min each and blotted using sterile blotting paper (Whatman Grade 3MM, Cytiva, Buckinghamshire, UK). The recovered wash water was plated on Nutrient agar and incubated for 24 h to confirm that surface disinfection was effective. It is noteworthy that surface disinfection reduces epiphyte levels but may not eliminate them.

### DNA metabarcoding of bacterial and fungal endophytes in leaves

#### DNA extraction and next generation sequencing of DNA from wrapping leaves

Leaf samples (2 g) were flash frozen in liquid nitrogen and grounded using a sterile mortar and pestle. According to the manufacturer’s instructions, DNA was extracted from each ground leaf sample (100 mg) using the DNeasy Plant Pro Kit (Qiagen, Hilden, Germany). The quality and concentration of the extracted DNA from all samples were measured using the Nanodrop One machine (Thermo Scientific, Waltham, MA, United States). For bacterial metabarcoding, 16S rRNA genes in distinct regions (16S V3-V4) were amplified with specific primers (e.g., 16S V4: 515F-806R), while for fungi, the ITS1/ITS2 region was amplified. All PCR mixtures contained 15 μL of Phusion High-Fidelity PCR Master Mix (New England Biolabs), 0.2 μM of each primer and 10 ng target DNA, and cycling conditions consisted of a first denaturation step at 98 °C for 1 min, followed by 30 cycles at 98 °C (10s), 50 °C (30s) and 72 °C (30s) and a final 5 min extension at 72 °C. For Library preparations, following the manufacturer’s recommendations, sequencing libraries were generated with NEBNext Ultra II DNA Library Prep Kit (Cat No. E7645). Library quality was evaluated using the Qubit@ 2.0 Fluorometer (Thermo Scientific, Waltham, MA, United States) and the Agilent Bioanalyzer 2100 (Agilent Technologies, Santa Clara, CA, United States). Finally, the library was sequenced on an Illumina NovaSeq platform, and 250 bp paired-end reads were generated.

#### Predicted functional analysis of bacteria and fungi in wrapping leaves

The functional analysis of bacteria and fungi in wrapping leaves was predicted with the aid of the PICRUSt2 package version 2.2.0^1^ (Douglas et al., 2020) in R version 4.4.3. The predicted functions were then evaluated and annotated based on the Kyoto Encyclopaedia of Genes and Genomes (KEGG) orthology (KO) pathways (Kanehisa et al., 2017). Only bacterial and fungal community functional genes contributing to pathogenicity and nutrient metabolism were investigated. Its noteworthy that functional inferences made by PICRUSt2 are predictions, hence cannot be used to conclude on the observed functionalities.

#### Statistical analysis

Sequenced data was analysed using appropriate pipelines QIIME2, and MicrobiotaProcess package in R.

## Results

### Alpha diversity of bacteria and fungi in wrapping leaves

**Figure 2:**
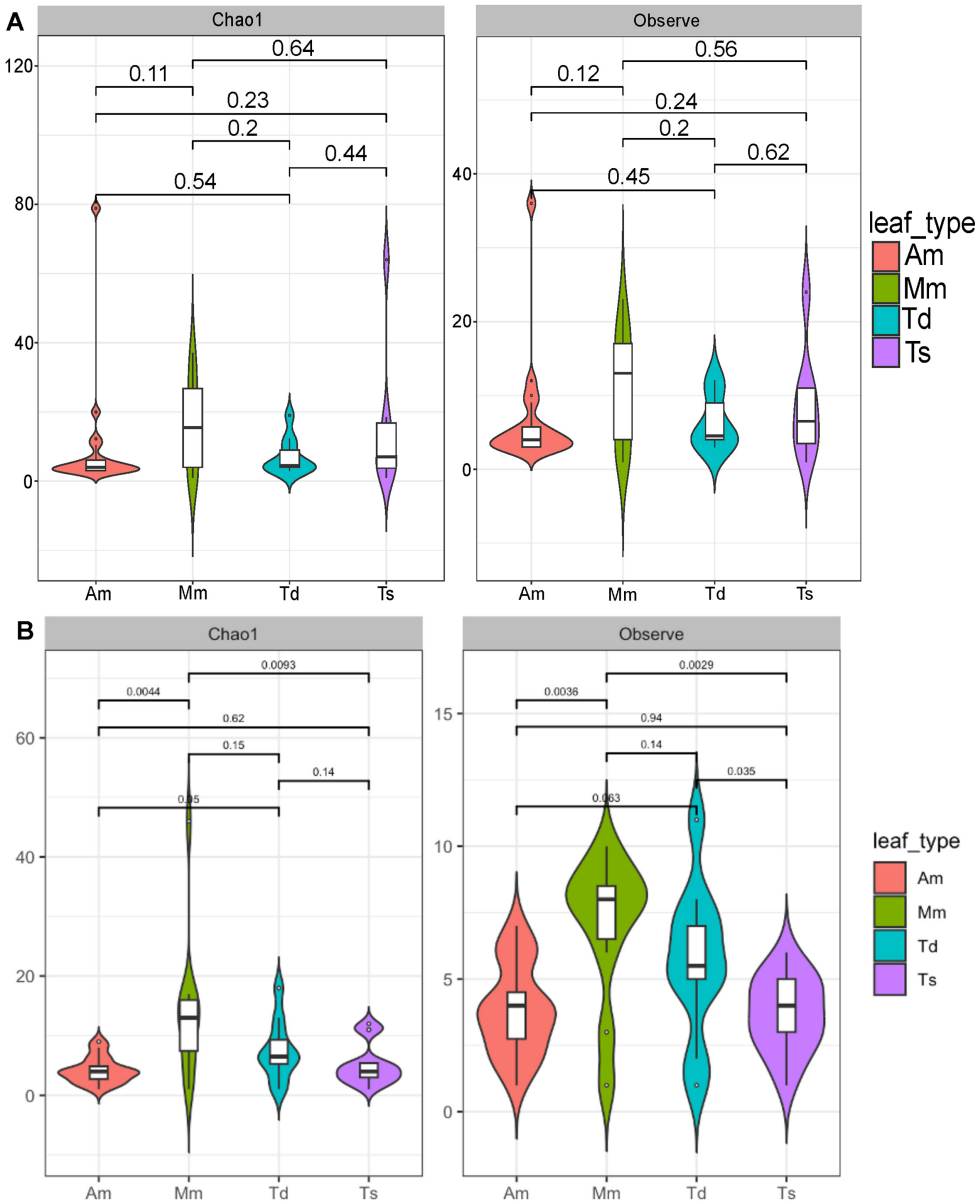
Bacterial **(A)** and fungal **(B)** diversities of wrapping leaves based on the Chao1 and observe index. *Alstonia macrophylla* (Am), *Megaphrynium macrostachyum* (Mm), *Thaumatococcus daniellii* (Td), and *Theobroma* species (Ts).

Results obtained from the bacterial alpha diversity (Chao1 index) revealed the highest estimated bacterial species richness (Range = 2–80) and variability in *Alstonia macrophylla* (Am). *Megaphrynium macrostachyum* (Mm) revealed a narrower range of values albeit a higher median (18) compared to *Thaumatococcus daniellii* (Td, median = 5) and *Theobroma* species (Ts, median = 8) which had lower medians and narrower ranges indicating a lower bacterial species richness (Figure 2A). However, the *p*-values obtained were not statistically significant across all the leaf types as all *p*-values obtained were >0.05. For the Observed index, the alpha diversity of bacterial species, all leaf types analysed revealed a similar pattern in terms of variability and species richness obtained in the Chao1 index. The median values (4–14) also revealed a similar pattern obtained in the Chao1 index (Figure 2A). Also, the *p*-values obtained were not statistically significant across all the leaf types as all *p*-values obtained were >0.05, however, Mm revealed higher median richness when compared to other leaf types.

Results obtained from the fungal alpha diversity (Chao1 index) revealed the highest estimated fungal species richness and variability in Mm. The samples *A. macrophylla* and *T. daniellii* showed lower median values, while *Theobroma* sp. had the smallest median value (median = 4) indicating low fungal diversity. A significantly higher fungal diversity was observed in *M. macrostachyum* samples when compared to *A. macrophylla* and *Theobroma* sp. at *p*-values < 0.05. There was no significant difference between Mm and Td (Figure 2B). For the Observed index, the alpha diversity of fungal species, *M. macrostachyum* samples revealed the highest species richness and variability followed closely by *T. daniellii*, *Theobroma* sp. and *A. macrophylla* samples, although *T. daniellii* samples revealed median values (6) slightly higher than *Theobroma* sp. and *A. macrophylla* samples (median = 4). In addition, *M. macrostachyum* samples were significantly higher in fungal diversity when compared to *A. macrophylla* and *Theobroma* sp. samples at *p*-values < 0.05. Also, *T. daniellii* was significantly higher in fungal diversity than *Theobroma* sp. samples at *p*-values < 0.05.

### Beta diversity of bacteria and fungi in wrapping leaves

**Figure 3:**
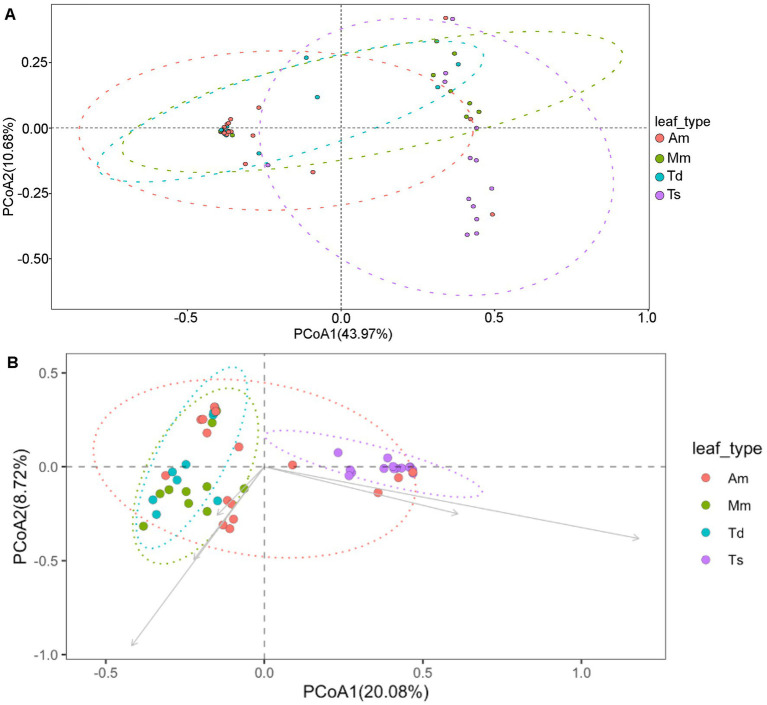
Principal coordinate analysis (PCoA) plot, showing the similarities in microbial beta diversity among wrapping leaves: **(A)** bacterial beta diversity; **(B)** fungal beta diversity using the Bray–Curtis dissimilarity. *Alstonia macrophylla* (Am), *Megaphrynium macrostachyum* (Mm), *Thaumatococcus daniellii* (Td), and *Theobroma species* (Ts).

Beta diversity analysis based on Bray–Curtis dissimilarity was carried out to quantitatively estimate and visualise microbial distribution within the wrapping leaves. The bacterial diversity in the wrapping leaves were grouped in the PCoA1 and PCoA2. On the x- axis, PCoA1 reveals an 43.97% while on the y-axis, PCoA2 reveals a 10.68% variation in bacterial composition in the wrapping leaves, respectively. Both axes reveal a good representation of the data with a total of 54.65% in bacterial variation (Figure 3A). The PERMANOVA test for the bacterial diversity revealed a significant difference between the four food wrapping leaves investigated.

The fungal diversity in the food wrapping leaves were also grouped in the PCoA1 and PCoA2. On the x-axis, PCoA1 reveals a 20.08% while on the y-axis, PCoA2 reveals an 8.72% variation in fungal composition in the wrapping leaves, respectively. Both axes reveal a good representation of the data with a total of 28.8% in fungal variation (Figure 3B). The fungal diversity also revealed a significant difference between the four food wrapping leaves investigated.

### Relative abundance of bacteria in wrapping leaves

**Figure 4:**
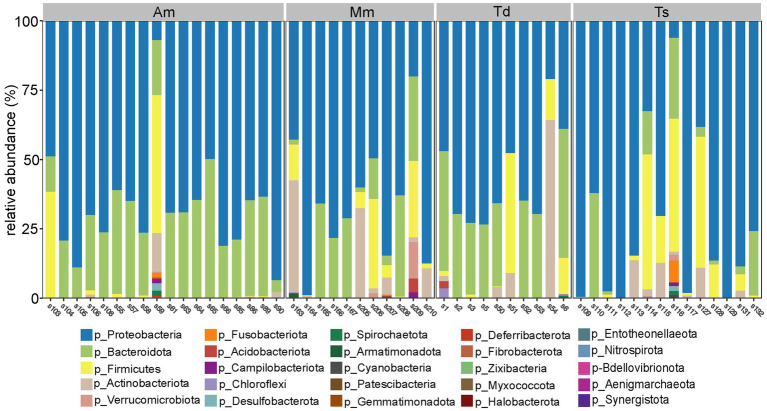
Relative abundance of bacteria from wrapping leaves at phylum level. *Alstonia macrophylla* (Am), *Megaphrynium macrostachyum* (Mm), *Thaumatococcus daniellii* (Td), and *Theobroma* species (Ts).

An overview of the bacteria communities in the leaf samples investigated revealed the dominance of the phyla *Proteobacteria* (5–100%) (Figure 4). This was followed by the *Bacteroidota* (5–100%) which revealed varying abundance across different samples. The *Firmicutes* (1–57%), the third most dominant phlya was widely distributed with significant variation across the samples. In a few samples, the *Actinobacteriota* and *Verrucomicrobiota* also feature across the samples. Other phyla detected sporadically include the *Chloroflexi*, *Fusobacteriota*, *Spirochaetota*, *Defferibacterota*, and *Acidobacteriota*. Based on the samples investigated, the *Proteobacteria* showed a consistent dominance in *A. macrophylla* and *Theobroma* sp. samples while the *Bacteroidota* and *Firmicutes* were relatively dominant in the *M. macrostachyum* and *T. daniellii* samples compared to the *A. macrophylla* and *Theobroma* sp. samples.

**Figure 5:**
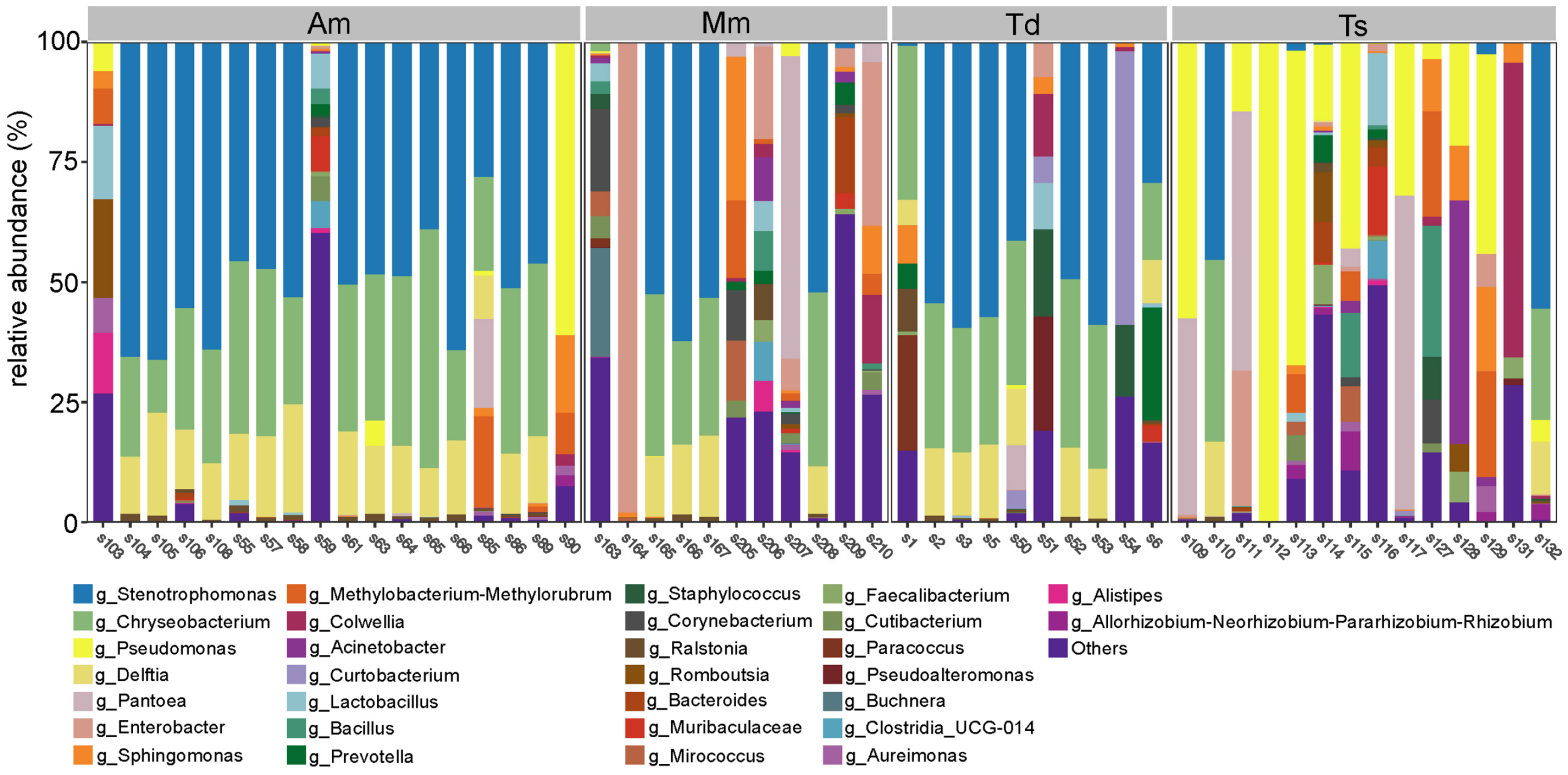
Relative abundance of bacteria from wrapping leaves at genus level. *Alstonia macrophylla* (Am), *Megaphrynium macrostachyum* (Mm), *Thaumatococcus daniellii* (Td), and *Theobroma* species (Ts).

Based on genera, the relative abundance of bacteria across the leaf samples were determined (Figure 5). Across the *A. macrophylla* samples, the dominant genus is the *Stenotrophomonas* (60–95%) followed by *Pseudomonas*, *Chryseobacterium*, and *Delftia.* Other less abundant genera include the *Enterobacter, Sphingomonas,* and *Methylobacterium-Methylorubrum*. The *M. macrostachyum* samples revealed increased bacterial diversity compared to *A. macrophylla*. However, *Stenotrophomonas* (30–70%) *was* still predominant. *Chryseobacterium*, *Acinetobacter*, *Lactobacillus*, *Bacillus*, and *Romboutsia* also revealed higher relative abundance. Anaerobic bacteria such as *Bacteroides*, *Muribaculaceae*, and *Micrococcus* were also detected. The *T. daniellii* samples showed a more heterogenous bacterial diversity. Despite *Stenotrophomonas* (55–90%) still dominating, other genera such as *Faecalibacterium*, *Cutibacterium*, *Paracoccus*, and *Buchnera* appear in higher relative proportions. The *Theobroma* sp. samples also showed diverse bacterial community with *Stenotrophomonas* (25–65%), *Pseudomonas*, and *Chryseobacterium* high highly abundant. Some samples also revealed high abundance of *Allorhizobium-Neorhizobium-Pararhizobium-Rhizobium*, *Ralstonia*, and *Corynebacterium*.

**Figure 6:**
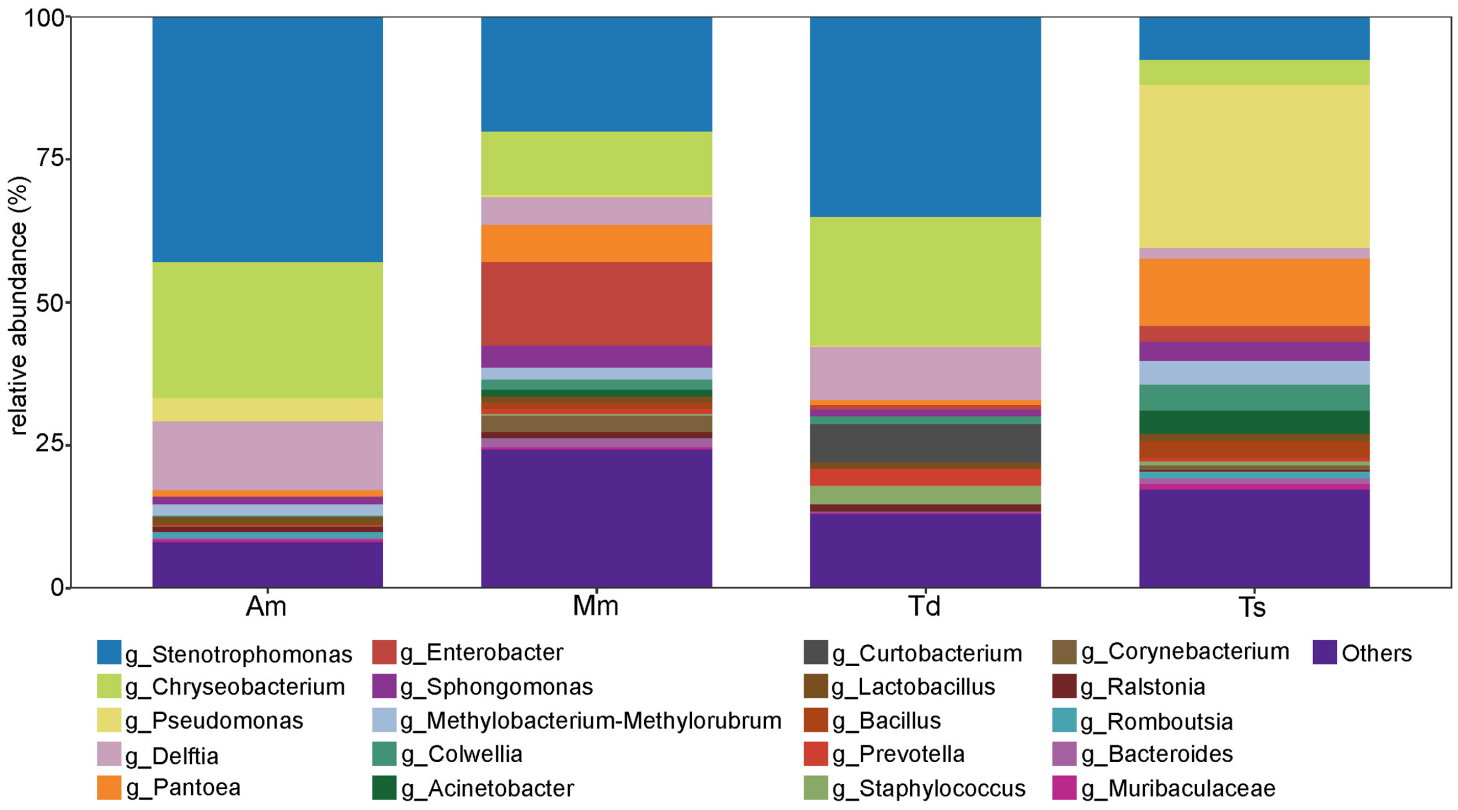
Top abundant 20 genera from wrapping leaves. *Alstonia macrophylla* (Am), *Megaphrynium macrostachyum* (Mm), *Thaumatococcus daniellii* (Td), and *Theobroma* species (Ts).

The top 20 dominant bacteria genera across the leaf samples was also determined (Figure 6). In *A. macrophylla* samples, *Stenotrophomonas* was strongly dominant followed by *Chryseobacterium* and *Pseudomonas*. *Delftia*, *Methylobacterium-Methylorubrum*, and *Sphingomonas* were also present however in lower relative abundances. The microbial diversity observed in *M. macrostachyum* samples were much higher than in *A. macrophylla*. *Stenotrophomonas* was still dominant however in reduced proportions when compared to *A. macrophylla*. *Chryseobacterium* and *Pseudomonas* still recorded significant abundance in *M. macrostachyum*. However, *Enterobacter*, *Acinetobacter*, *Lactobacillus*, *Bacillus*, and *Bacteroides* also contribute to the microbial structure in *M. macrostachyum*. In *T. daniellii* samples, revealed a microbial diversity similar to *M. macrostachyum* with *Stenotrophomonas* remaining a major component. There was also an increased abundance of *Pseudomonas*, *Chryseobacterium*, and *Bacteroides* compared to *M. macrostachyum*. Additional genera such as *Ralstonia*, *Prevotella*, and *Muribaculaceae* are present in moderate proportions was observed. In *Theobroma* sp. samples, a similar bacterial composition to *A. macrophylla* was observed, with *Stenotrophomonas* as the dominant genus. *Chryseobacterium* and *Pseudomonas* were also prevalent in *Theobroma* sp. samples. Other genera such as *Bacillus*, *Acinetobacter*, and *Enterobacter* were also found in relatively lower proportions.

**Figure 7:**
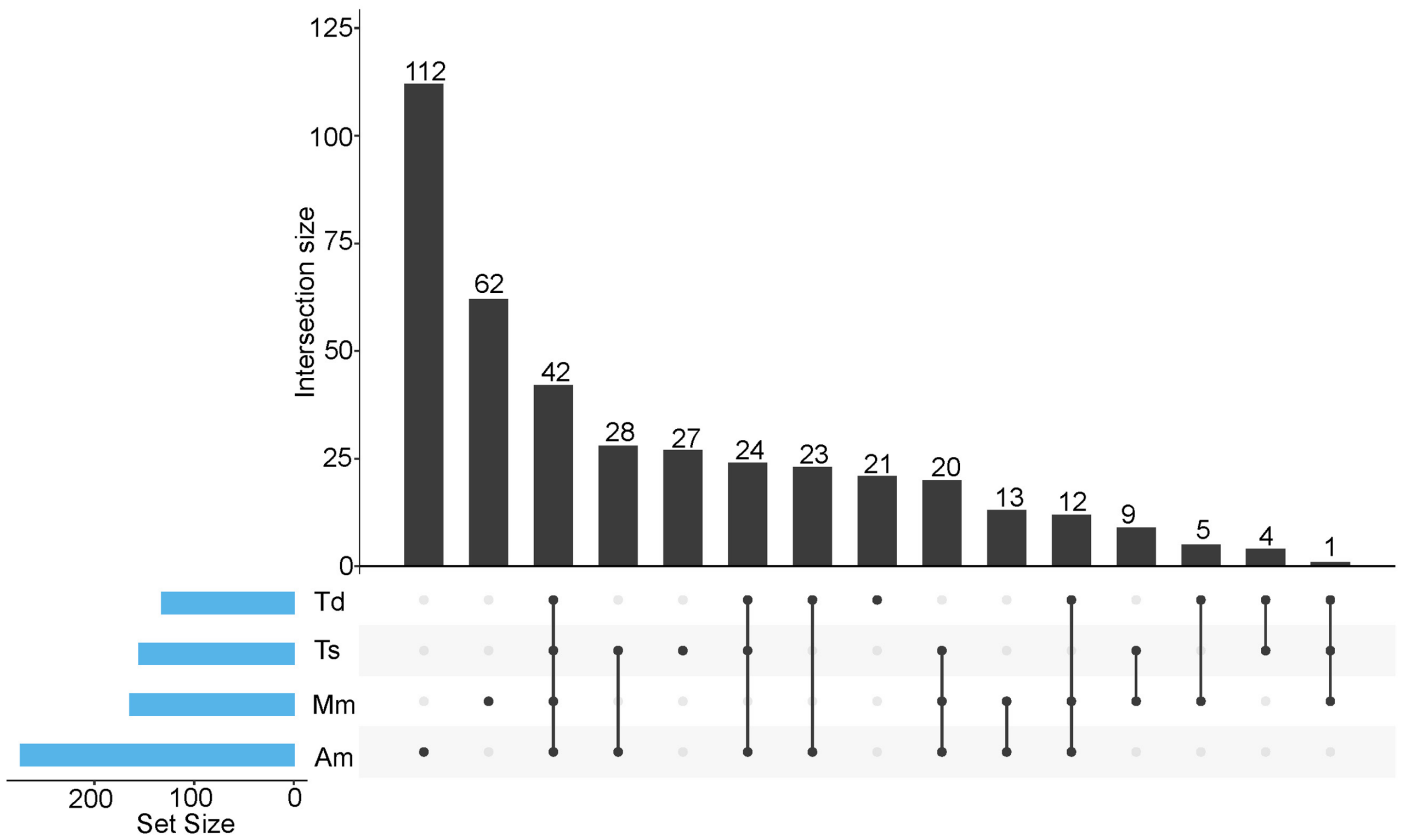
Upset plot showing shared and unique bacteria taxa in wrapping leaves. *Alstonia macrophylla* (Am), *Megaphrynium macrostachyum* (Mm), *Thaumatococcus daniellii* (Td), and *Theobroma* species (Ts).

The unique and shared bacteria taxa observed in the leaves investigated are represented in Figure 7. The largest vertical bar with 112 taxa represents bacterial taxa belonging to *A. macrophylla* closely followed by *M. macrostachyum* with 62 taxa, *Theobroma* sp. with 27 taxa and *T. daniellii* with 21 taxa. A total of 42 taxa was observed in all leaf samples, 24 taxa was observed in *A. macrophylla*, *Theobroma* sp. and *T. daniellii* samples, 20 taxa was observed in *A. macrophylla*, *M. macrostachyum* and *Theobroma* sp. samples, 12 taxa was observed in *A. macrophylla*, *M. macrostachyum* and *T. daniellii* samples while 1 taxon was observed in *M. macrostachyum*, *Theobroma* sp. and *T. daniellii* samples. Also, *A. macrophylla* and *Theobroma* sp. samples shared 28 taxa, 23 taxa were observed in *A. macrophylla* and *T. daniellii* samples, 13 taxa were observed in *A. macrophylla* and *M. macrostachyum* samples, 9 taxa were observed in *M. macrostachyum* and *Theobroma* sp. samples while 4 taxa were observed in *Theobroma* sp. and *T. daniellii* samples.

### Relative abundance of fungi in leaves

**Figure 8:**
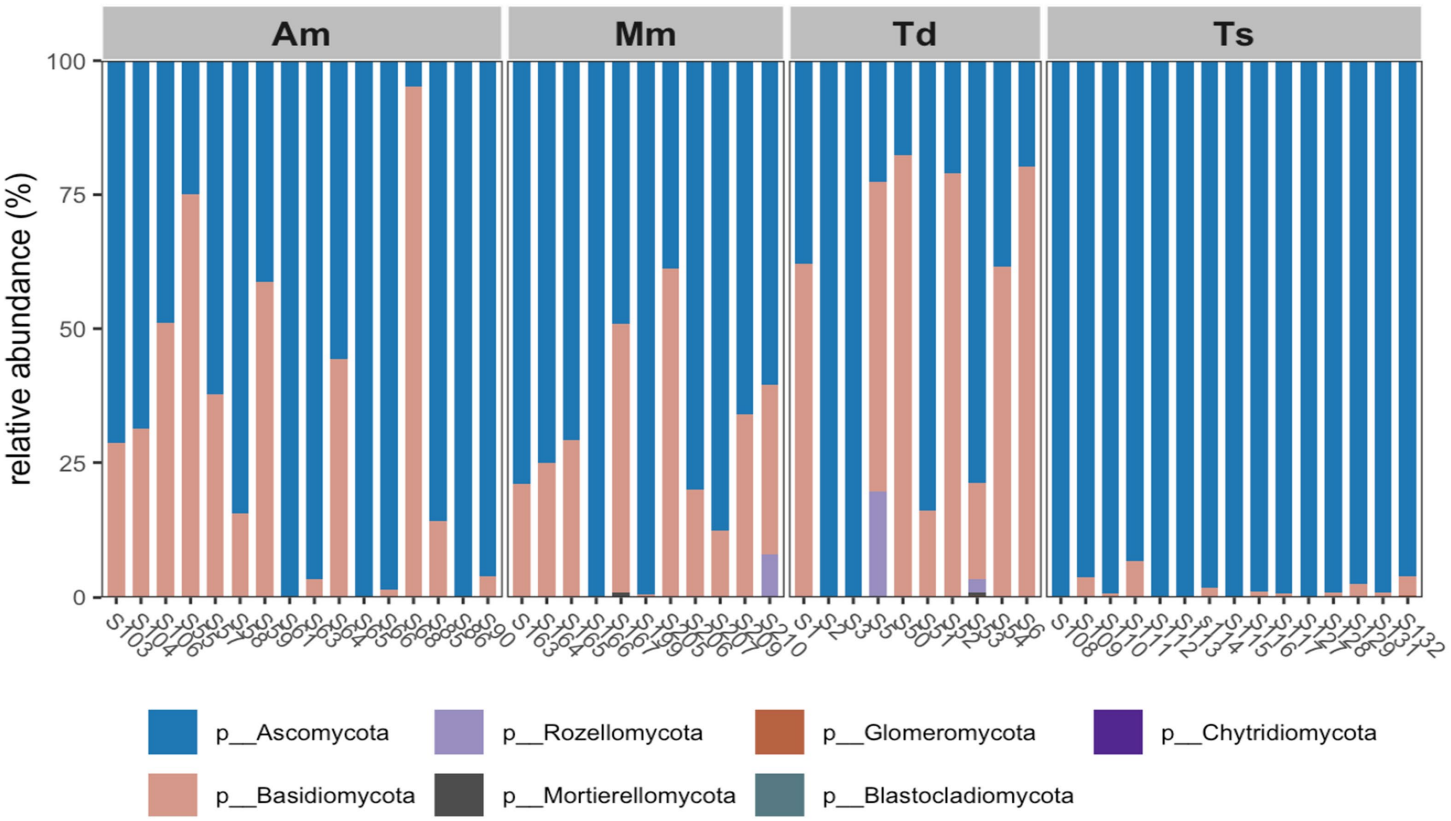
Relative abundance of fungi from wrapping leaves at phylum level. *Alstonia macrophylla* (Am), *Megaphrynium macrostachyum* (Mm), *Thaumatococcus daniellii* (Td), and *Theobroma* species (Ts).

An overview of the fungal communities in the wrapping leaves investigated revealed the dominance of the phyla *Ascomycota* with about 90% of the fungal community in *Alstonia macrophylla* and *Theobroma* species samples, closely followed by the *Basidiomycota* (80%) which was prominent in *Alstonia macrophylla*. *Megaphrynium macrostachyum*, *Thaumatococcus daniellii* samples. The Rozellomycota (10%) and Mortierellomycota (<5%) were the least abundant fungus observed particularly in *Megaphrynium macrostachyum* and *Thaumatococcus daniellii* samples (Figure 8).

**Figure 9:**
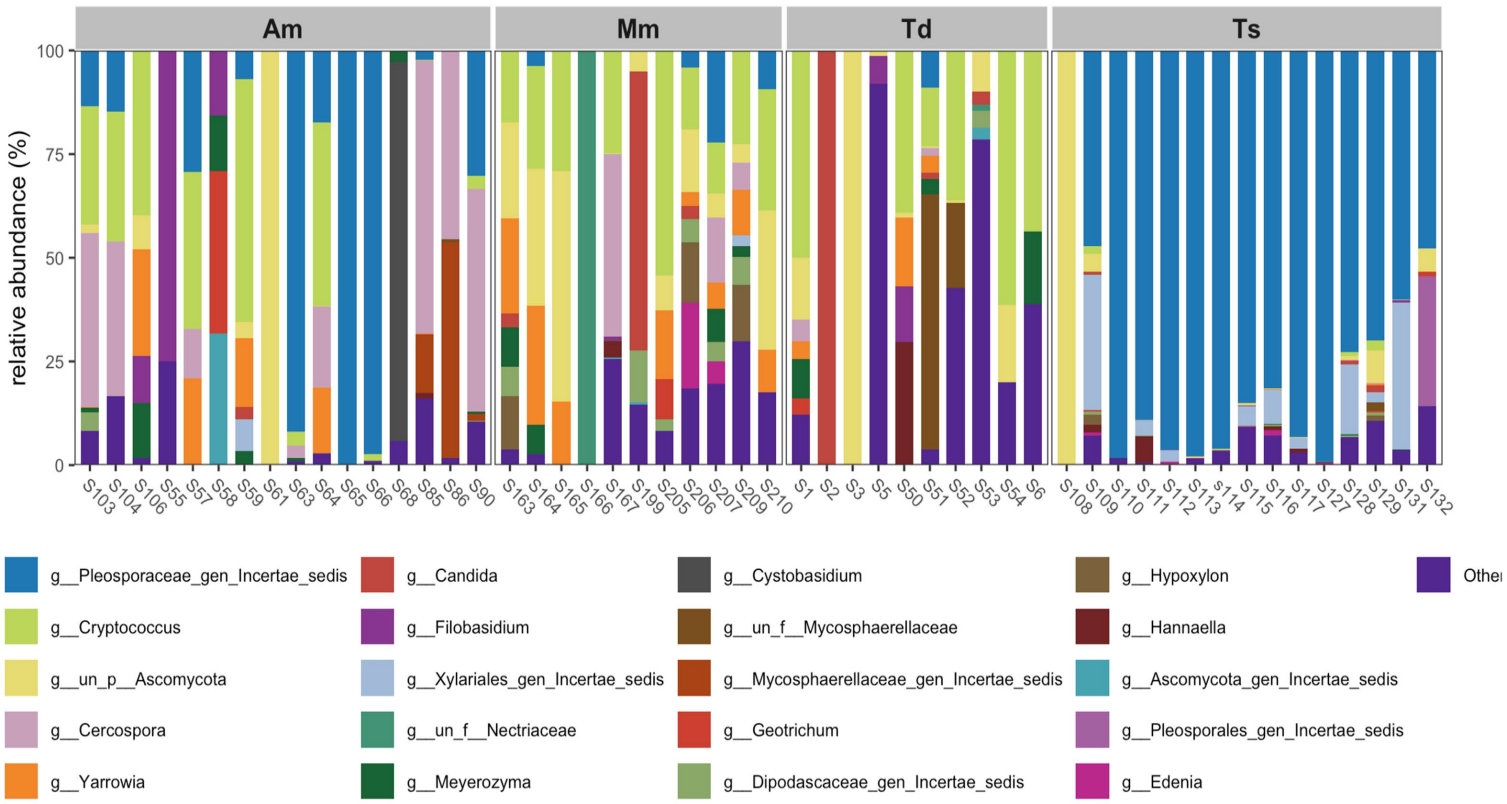
Relative abundance of fungi from wrapping leaves at genus level. *Alstonia macrophylla* (Am), *Megaphrynium macrostachyum* (Mm), *Thaumatococcus daniellii* (Td), and *Theobroma* species (Ts).

Based on genera, the relative abundance of fungi across the wrapping leaves were determined (Figure 9). The samples investigated reveals significant variation in fungal composition at the genus level. The *Theobroma* species samples revealed the least fungal diversity observed in all the samples investigated. *Pleosporaceae*_gen_*Incertae*_*sedis* revealed the highest relative abundance (about 100%) in the *Theobroma* species samples. Other less abundant genera observed in *Theobroma* species samples include, *Xylariales* gen_*Incertae*_*sedis*, *Ascomycota*, *Cercospora*, *Geotrichum*, *Candida*, *Yarrowia* and *Cryptococcus*. The *Thaumatococcus daniellii* samples revealed high fungal diversity when compared to *Theobroma* species. In *Thaumatococcus daniellii* samples, dominance by various genus was observed notably in *Candida* (0–5%), *Ascomycota* (40–55%), *Hypoxylon* (5–10%) and *Cryptococcus* (0–5%). All *Thaumatococcus daniellii* samples showed distinct composition. The *Megaphrynium macrostachyum* and *Alstonia macrophylla* samples investigated also showed high fungal diversity revealing multiple genera with significant abundance. They include *Ascomycota* (35–45%), *Cercospora* (5–10%), *Candida* (5–8%)*, Cryptococcus* (5–10%)*, Pleosporaceae*_gen_*Incertae*_*sedis* (10–15%)*, Filobasidium* (25–35%) and *Geotrichum* (0–3%).

**Figure 10:**
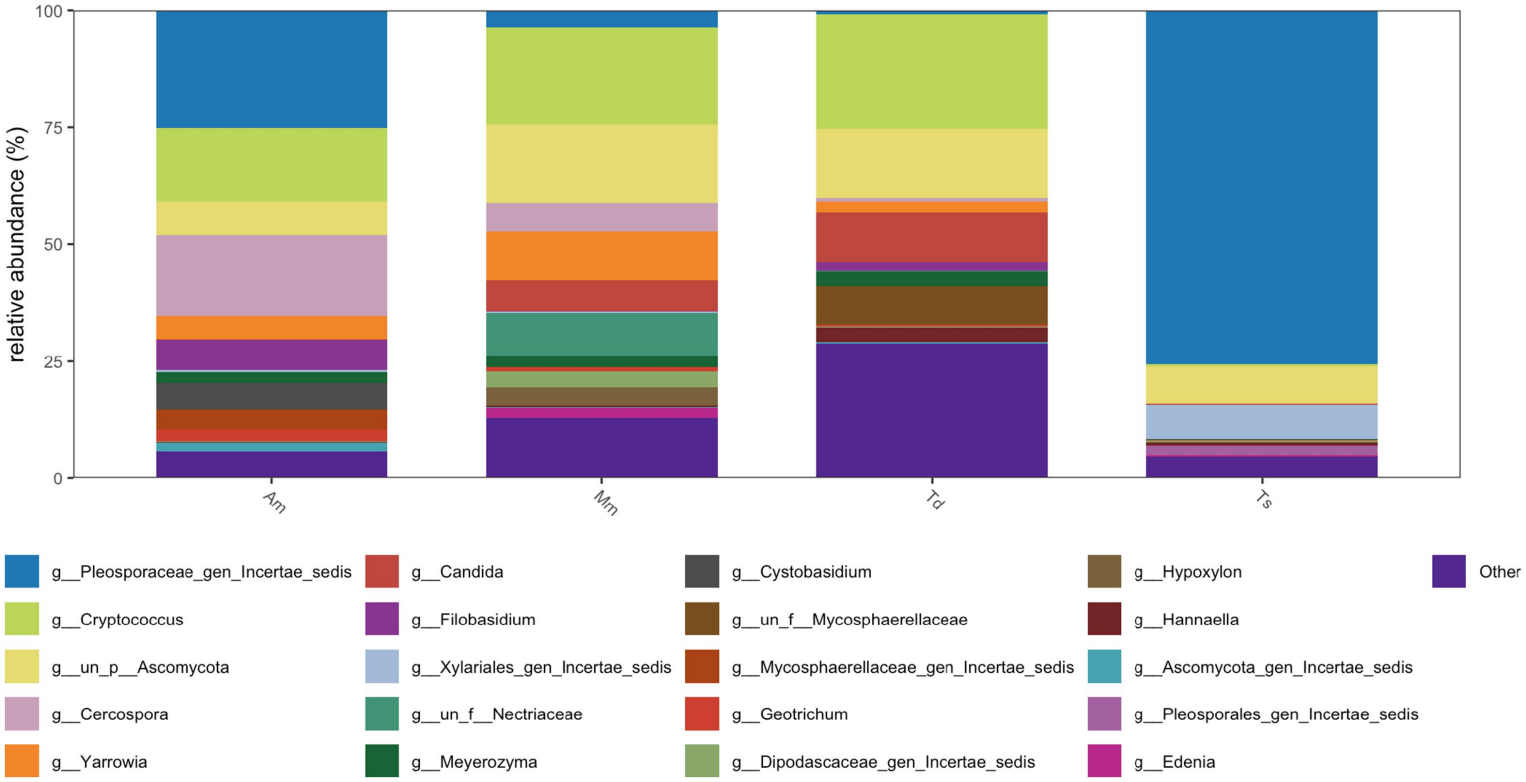
Top abundant 20 genera from wrapping leaves. *Alstonia macrophylla* (Am), *Megaphrynium macrostachyum* (Mm), *Thaumatococcus daniellii* (Td), and *Theobroma* species (Ts).

The top 20 dominant bacteria genera across the leaf samples was also determined (Figure 10). In Am samples, multiple genera contribute to its huge diversity. Notable genera which include: *Pleosporaceae*_gen_*Incertae*_*sedis, Cercospora* and *Cryptococcus* were observed. There was no clear dominance by a single genus highlighting an even fungal distribution. In *Megaphrynium macrostachyum* samples, diverse fungal genera were also observed. Key genera observed include: *Cryptococcus*, *Ascomycota* and *Yarrowia.* In *Thaumatococcus daniellii* samples, fungal diversity is less than *Alstonia macrophylla* and *Megaphrynium macrostachyum* samples. Notable genera observed include: *Cryptococcus*, *Ascomycota* and *Candida*.

**Figure 11:**
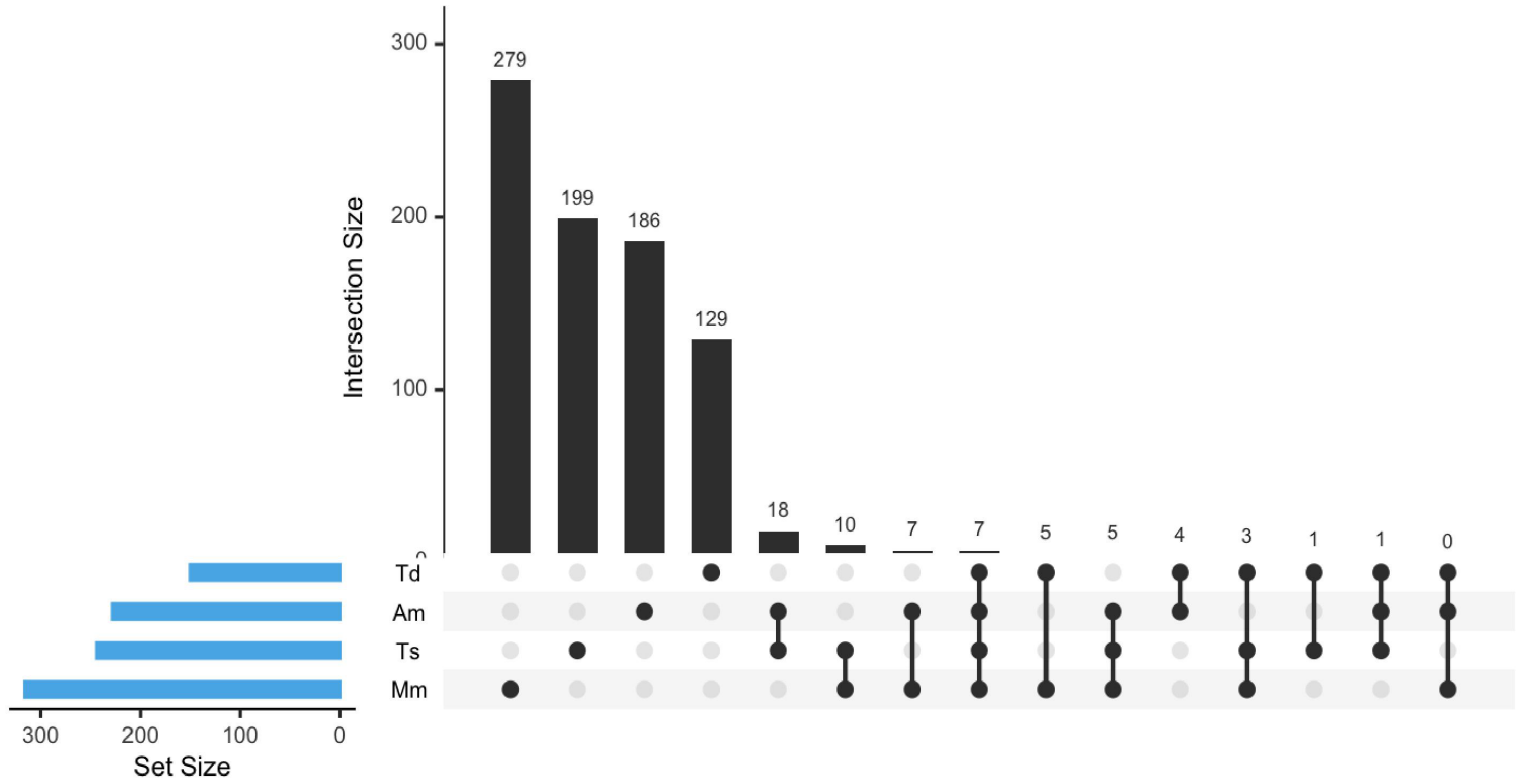
Upset plot showing shared and unique fungal taxa in wrapping leaves. *Alstonia macrophylla* (Am), *Megaphrynium macrostachyum* (Mm), *Thaumatococcus daniellii* (Td), and *Theobroma* species (Ts).

The unique and shared fungal taxa observed in the leaves investigated are represented in Figure 11. The largest vertical bar with 279 taxa represents fungal taxa belonging to *Megaphrynium macrostachyum* closely followed by *Theobroma* species with 199 taxa, Am with 186 taxa and *Thaumatococcus daniellii* with 129 taxa. A total of 7 taxa was observed in all leaf samples, 5 taxa was observed in *Alstonia macrophylla*, *Theobroma* species and *Megaphrynium macrostachyum* samples, 3 taxa was observed in Mm, Ts and Td, while 1 taxon was observed in *Alstonia macrophylla*, *Theobroma* species and *Thaumatococcus daniellii* samples. In addition, *Alstonia macrophylla* and *Theobroma* species samples shared 18 taxa, 10 taxa were observed in *Theobroma* species and *Megaphrynium macrostachyum* samples, 7 taxa were observed in *Alstonia macrophylla* and *Megaphrynium macrostachyum* samples, 5 taxa were observed in *Megaphrynium macrostachyum* and *Thaumatococcus daniellii* samples, 4 taxa were observed in *Alstonia macrophylla* and *Thaumatococcus daniellii* samples while 1 taxon was observed in *Theobroma* species and *Thaumatococcus daniellii* samples.

### Predicted functional properties of bacteria in wrapping leaves

**Figure 12:**
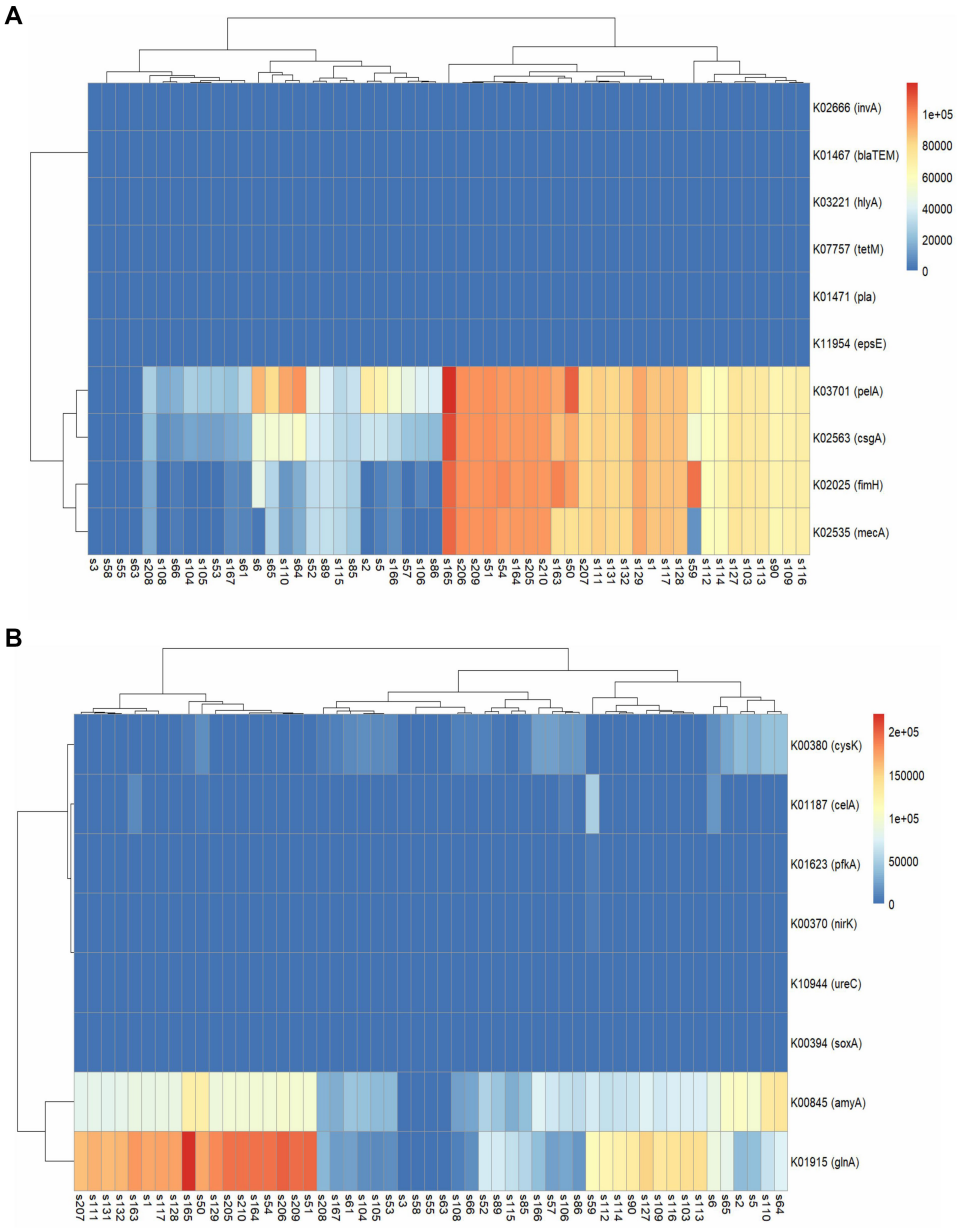
**(A)** Abundance of KEGG Orthology (KO) terms associated with nutrient metabolic pathways. **(B)** Abundance of KEGG Orthology (KO) terms associated with pathogenic pathways.

A total of 71 predicted functions were observed from the KEGG orthology (KO) identifiers analysed. However, 9 significant functions relating to pathogenicity in humans (*n* = 3) and synthesis of secondary metabolites (*n* = 6) were determined. Functions associated with pathogenicity include: the ability to cause infection by *Escherichia coli*, *Shigella* sp. and *Vibrio cholerae* observed in *Alstonia macrophylla*, *Megaphrynium macrostachyum* and *Theobroma* species samples, respectively. Genes associated with antibiotic resistance (K01467 (*blaTEM*), K02535 (*mecA*), K07757 (*tetM*)) as well as biofilm formation (K11954 (*epsE*), K03701 (*pelA*), K02563 (*csgA*)) were identified. There was also detection of genes associated with functions such as carbohydrate metabolism (K00845 (*amyA*), K00370 (*nirK*), K00380 (*cysK*), K01187 (*celA*), K01623 (*pfkA*)); Nitrogen metabolism (K00370 (*nirK*), K10944 (*ureC*), K01915 (*glnA*)); as well as sulphur metabolism (K00380 (*cysK*); K00394 (*soxA*)) (Figure 12A). Functions associated with metabolism and biosynthesis of secondary metabolites such as clavulanic acid, flavone, flavonol, isoflavonoid, monoterpenoid, indole alkaloid and O-glycan. From the aforementioned functions, only clavulanic acid was observed in *Megaphrynium macrostachyum* samples, while others were observed in *Alstonia macrophylla* samples. Clustering of genes into distinct groups (Figures 12A,B) suggests shared metabolic profiles among subsection of samples.

### Predicted functional properties of fungi in wrapping leaves

**Figure 13:**
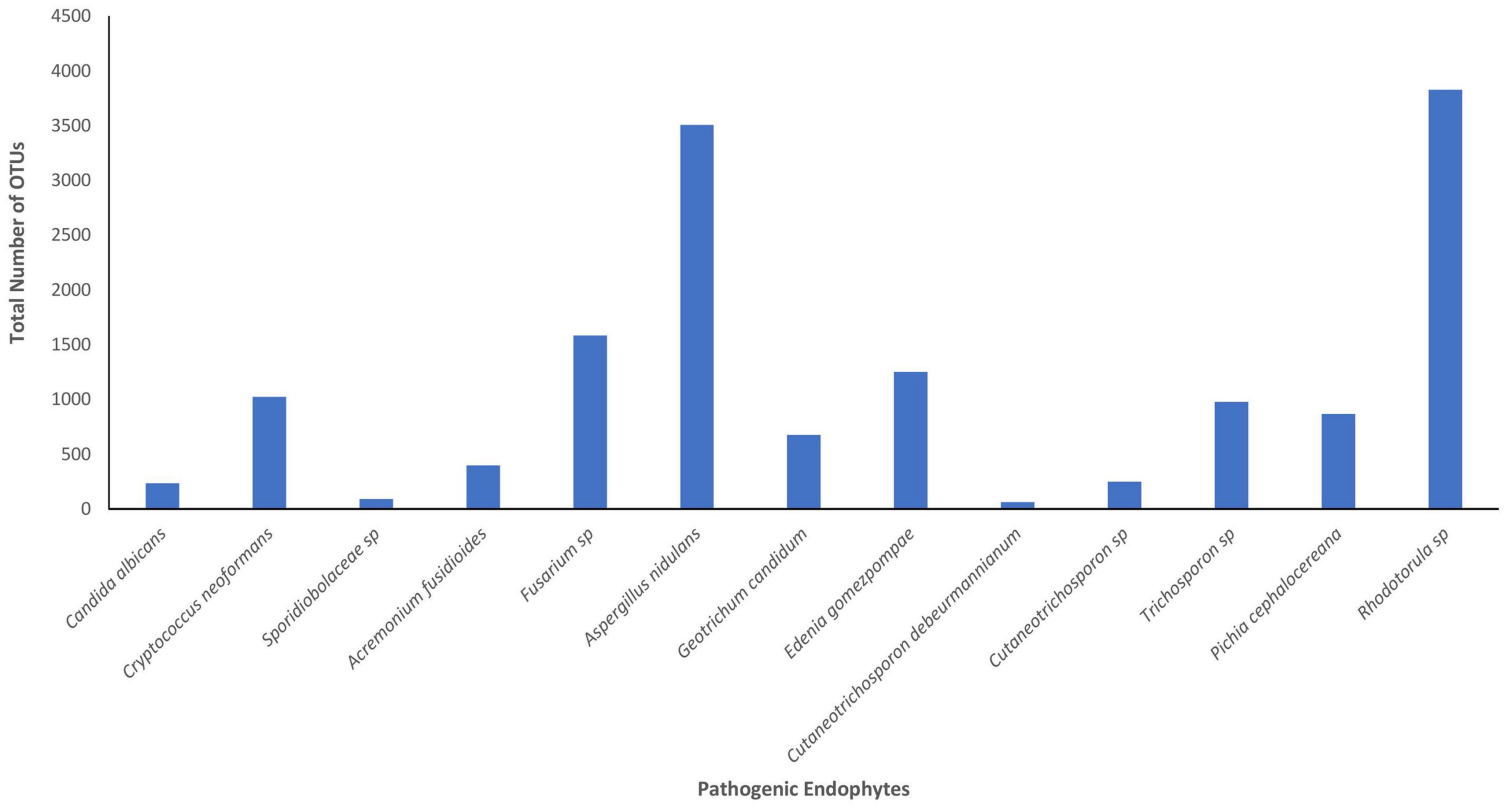
Number of OTUs of pathogenic fungi from wrapping leaves.

A total of 12 genera of pathogenic fungi (i.e., yeast-like, dimorphic and filamentous fungi) were observed in the wrapping leaves (Figure 13). The highest number of OTUs was observed in *Rhodotorula* sp. with 3,830 while the least was recorded in *Cutaneotrichosporon debeurmannianum* with 63. Their predicted functional roles include being animal pathogens (i.e., *Candida albicans, Cryptococcus neoformans*, *Fusarium* sp. and *Sporidiobolaceae* sp). Also, *Acremonium fusidioides, Aspergillus nidulans, Cutaneotrichosporon debeurmannianum*, *Cutaneotrichosporon* sp. and *Rhodotorula* sp. revealed roles as plant pathogens, emerging pathogens and putative endophytes. *Pichia cephalocereana* played roles animal and plant pathogens while *Geotrichum candidum* and *Edenia gomezpompae* played roles as animal pathogen and endophyte.

## Discussion

The long-standing global practise of using plant leaves for food packaging has gained renewed attention amid the current emphasis on environmental sustainability. These leaves are recognised for their accessibility, cost-effectiveness, and biodegradability. Furthermore, they contain phytochemicals and produce metabolites that can influence the shelf life and quality of packaged food. Diversity and composition of endophytic bacterial communities can vary across different plant species, with leaves often harbouring more bacterial species compared to the stem and roots (Emitaro et al., 2024). Dominant endophytic bacterial genera identified in this study include *Stenotrophomonas*, *Pseudomonas*, *Bacillus* etc., which have been reported as core endophytic microbiome across a wide range of plant species (Costa et al., 2012). The distinct bacterial community profile identified in this study suggests a difference in the abundance and association of common putative endophytes, possibly driven by plant-specific factors. Such a difference in abundance of leaf endophytic communities have been directly linked to the health status of plant leaves (Li et al., 2024). Within these plant leaves reside endophytic microbial communities, which, despite their presence, have not been thoroughly explored. The advent of next-generation sequencing technologies has begun to reveal the substantial microbial diversity present within these plant matrices. Specifically, the diversity of putative bacterial and fungal endophytes in wrapping leaves such as *Alstonia macrophylla*, *Megaphrynium macrostachyum*, and *Thaumatococcus daniellii* is largely unexplored, although some studies exist on the microbial diversity of *Theobroma cacao* and *Theobroma grandiflorum* leaves (Barreto et al., 2008; Wemheuer et al., 2020; de Matos et al., 2024).

This study utilised 16S rRNA and ITS metabarcoding to investigate the diversity and predicted functional potential of endophytic bacterial and fungal communities associated with four commonly used food wrapping leaves: *Thaumatococcus daniellii*, *Alstonia macrophylla*, *Theobroma* species, and *Megaphrynium macrostachyum*. The findings indicated higher bacterial than fungal diversity in these wrapping leaves. The study also revealed that both bacterial and fungal taxa had both shared and unique compositions across the different types of wrapping leaves examined.

Our analysis of alpha diversity revealed variations in microbial richness and variability among the different leaf types. Bacterial species richness and variability (Chao1 and Observed indices) were highest in *Alstonia macrophylla* samples, although these differences were not statistically significant across all leaf types examined. In contrast, fungal species richness and variability were highest in *Megaphrynium macrostachyum* samples, with significantly higher diversity than in *Alstonia macrophylla* and Theobroma species samples, as indicated by both Chao1 and Observed indices. *Thaumatococcus daniellii* also exhibited significantly higher fungal diversity than *Theobroma* species based on the Observed index. Conserved alpha diversity across different leaf types suggests a conserved/stable core microbiome or shared dispersal of putative core bacterial endophytes. This could be due to the recruitment of endophytes from the same regional core microbial pool rather than host-specific selection (Durán, 2024).

Beta diversity analysis, utilising Bray–Curtis dissimilarity and visualised through Principal Coordinates Analysis (PCoA), further supported the notion of distinct microbial communities associated with each leaf type. Bacterial communities showed a notable separation, with PCoA1 and PCoA2 accounting for 54.65% of the bacterial variation, and PERMANOVA confirmed a significant difference among the four-leaf types. Fungal communities also showed differentiation, with PCoA1 and PCoA2 explaining 28.8% of the fungal variation. This clear separation in both bacterial and fungal community composition across the different leaf species emphasises the unique microbial ecosystems hosted by each plant type, likely influenced by factors such as plant physiology, habitat, and interactions with the surrounding environment (Trivedi et al., 2020).

Detailed taxonomic analysis provided insight into the dominant microbial groups. For bacteria, Proteobacteria was consistently the most dominant phylum across the samples. Other prevalent phyla included Bacteroidota and Firmicutes, with varying abundance depending on the leaf type. At the genus level, *Stenotrophomonas* was identified as the dominant bacterial genus across multiple leaf types, although its relative abundance varied. *Stenotrophomonas* can persist on leaves via biofilm formation and production of enzymes (proteases and lipases) that could compromise the quality and texture of plant-based foods (Li et al., 2019). Other frequently observed genera included *Pseudomonas*, *Chryseobacterium, Acinetobacter*, *Lactobacillus*, *Bacillus*, *Bacteroides*, *Muribaculaceae*, *Faecalibacterium*, and *Enterobacter*. The dominance of Proteobacteria and genera such as *Pseudomonas* is consistent with findings in other plant endosphere studies (Niem et al., 2020; Xia et al., 2021). However, the high prevalence of *Stenotrophomonas* and the presence of genera such as *Faecalibacterium*, *Bacteroides*, and *Muribaculaceae*, which are typically associated with animal or human gut environments, warrants further investigation into their origin and potential implications in the leaf endosphere (Sessitsch et al., 2023; Zhang et al., 2023).

In terms of fungal communities, Ascomycota was the dominant phylum, particularly prevalent in *Alstonia macrophylla* and *Theobroma* species, while Basidiomycota was also prominent across *Alstonia macrophylla*, *Megaphrynium macrostachyum*, and *Thaumatococcus daniellii* samples. At the genus level, *Pleosporaceae*_gen_Incertae_sedis was the most abundant genus, especially in *Theobroma* species. Other significant fungal genera detected included Candida, Ascomycota (likely unidentified genera within this phylum), *Hypoxylon*, *Cryptococcus*, *Cercospora*, *Geotrichum*, *Yarrowia*, and *Filobasidium*. The high diversity of fungal genera observed, particularly in *Thaumatococcus daniellii*, *Megaphrynium macrostachyum*, and *Alstonia macrophylla*, suggests a complex fungal endosphere (Halecker et al., 2020; Grabka et al., 2022; Liu et al., 2022; Yang et al., 2023; Sena et al., 2024; Li et al., 2025).

A core microbiome analysis revealed a set of microorganisms shared across all examined leaf types. Specifically, 42 bacterial and 7 fungal taxa were common to *Alstonia macrophylla*, *Megaphrynium macrostachyum*, *Theobroma* species, and *Thaumatococcus daniellii*. The unique bacterial taxa associated with *A. macrophylla* may be related to its ability to produce leaves with prolonged longevity, thereby increasing the period for microbial colonisation. Its large surface area also provides more entry points and enhanced habitat for potential recruitment/establishment of endophytic bacteria, as opposed to *Thaumatococcus daniellii,* with reduced surface area and short-lived leaves (Penuelas et al., 2012; Lan et al., 2024). This core community might represent a stable endophytic consortium capable of colonising diverse plant hosts. Additionally, each leaf type harboured a number of unique taxa, contributing to the observed differences in beta diversity (Castillo-González et al., 2024).

The predicted functional analysis, performed using PICRUSt2 based on KEGG orthology, aimed to infer the potential metabolic capabilities and pathogenicity-related traits of the bacterial and fungal communities. While a large number of predicted functions were identified, focus was placed on those related to pathogenicity and nutrient metabolism. Predicted bacterial functions included pathways associated with the ability to cause infection by specific pathogens, such as *Escherichia coli*, *Shigella* sp., and *Vibrio cholerae*, inferred from samples of *Alstonia macrophylla*, *Megaphrynium macrostachyum*, and *Theobroma* species (Søborg et al., 2016). The prediction for genes associated with pel polysaccharide biosynthesis (*pelA*, K03701) and exopolysaccharide synthesis (*epsE*, K11954) indicates a capacity for biofilm formation among the microbial communities (Whitfield et al., 2020). Of particular concern is the co-occurrence of antibiotic resistance genes, including β-lactamase (*blaTEM*, K01467), methicillin resistance (*mecA*, K02535), and tetracycline resistance (*tetM*, K07757), which raises significant public health implications (Jian et al., 2021). Furthermore, the inferred metabolic potential for carbohydrate, nitrogen, and sulphur metabolism highlights the potential functional adaptability of these microbes in nutrient acquisition and energy production, emphasising their environmental versatility (Baritugo et al., 2018; Zhang et al., 2019). Furthermore, the potential to synthesise secondary metabolites, such as clavulanic acid (in Megaphrynium macrostachyum) and various flavonoids and alkaloids (in *Alstonia macrophylla*), was predicted. In fungi, the analysis identified 12 genera potentially pathogenic to humans. These included genera known to be animal pathogens (*Candida albicans*, *Cryptococcus neoformans*, *Fusarium* sp., *Sporidiobolacea*e sp.), plant pathogens/ putative endophytes (*Acremonium fusidioides*, *Aspergillus nidulans*, *Cutaneotrichosporon* spp., *Rhodotorula* sp.), or even both animal and plant pathogens (*Pichia cephalocereana*). The presence of these potentially harmful taxa within the leaves underscores a potential public health risk associated with their use in direct contact with food (Akinsemolu and Onyeaka, 2024; Bastos et al., 2020; Cespedes et al., 2022; Igere, 2024; Kamilari et al., 2023; Posso et al., 2024; Simões et al., 2023; Steenwyk et al., 2020; Tsakem et al., 2024; Zhou et al., 2009).

It is important to note that these functional assignments are predictions based on genetic markers and metabarcoding data. They do not necessarily indicate that these functions are actively being expressed or that these organisms are viable pathogens in this context. Further experimental validation, such as the isolation and characterisation of specific strains, viability assessment, and targeted functional assays (e.g., pathogenicity tests, metabolite analysis) (Ferone et al., 2020; Trinh and Lee, 2022), is required to confirm these predicted capabilities and fully assess the safety implications.

Despite the potential risks highlighted by predicted pathogenicity functions, the identified communities likely also contain endophytes with beneficial properties. For instance, taxa detected in the endosphere, such as *Lactobacillus*, might contribute to food fermentation or produce compounds that enhance food quality or preservation (Sanchez and Demain, 2017), supports the possibility of beneficial endophyte roles.

## Conclusion

This study provides a preliminary snapshot of the diverse and distinct endophytic bacterial and fungal communities inhabiting various food wrapping leaves used in Nigeria. While revealing potentially beneficial microbial components and emphasising the complexity of the endosphere, the study also highlights taxa with predicted pathogenic capabilities. These findings stress the need for further research to move beyond prediction to functional validation, ultimately aiming to identify leaf varieties with favourable microbiological profiles through evidence-based screening, while preserving the cultural, ecological, and economic value of traditional leaf-based food packaging. The comprehensive microbial profiles established here serve as a foundational resource for future targeted investigations into the specific roles and interactions of these putative endophytes within the context of food packaging.

## Data Availability

The original contributions presented in the study are publicly available. This data can be found at: https://www.ncbi.nlm.nih.gov/sra/PRJNA1449066.

## Footnotes

1
https://github.com/picrust/picrust2
