# Correction: Diversity and functional potential of bacterial and fungal endophytes in traditional food wrapping leaves reveal implications for artisanal food safety and quality

**DOI:** 10.3389/fmicb.2026.1881167

**Published:** 2026-06-23

**Authors:** Rasheed A. Adeleke, Thabang M. E. Machailoe, Michelle Malemagovha, Oluwaseyi S. Olanrewaju, Kazeem A. Alayande, Linda U. Obi, Oluwadamilola M. Makinde

**Affiliations:** Unit for Environmental Sciences and Management, North-West University (Potchefstroom Campus), Potchefstroom, South Africa

**Keywords:** bacteria, endophytes, food safety, fungi, ready-to-eat foods, wrapping leaves

There was a mistake in Figures 2, 3, 4, 5, 6, 7, 11, 12A, 12B, and 13 as published.

The high-quality figures were not utilized in the published version.

The corrected figures appear below.

**Figure 2:**
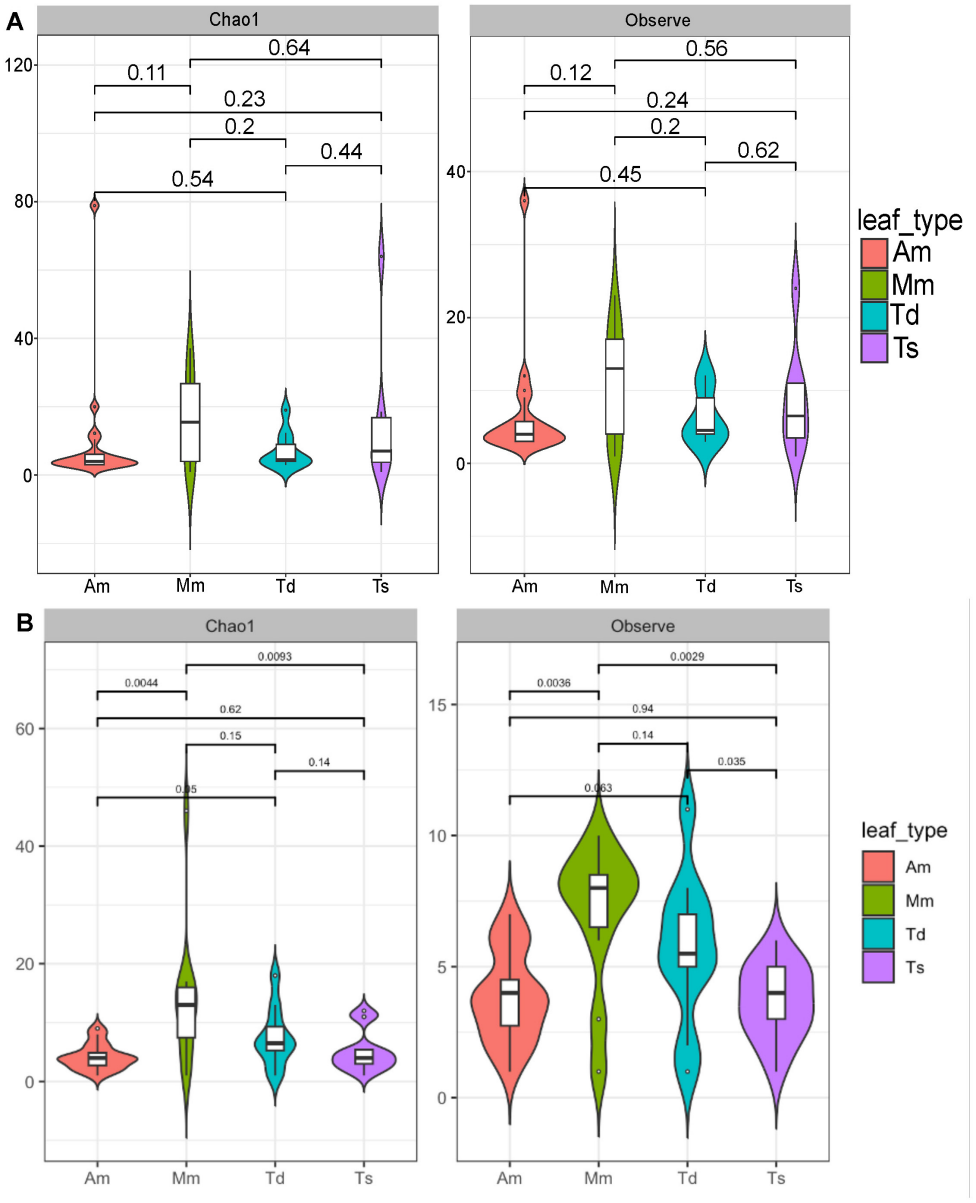
Bacterial **(A)** and fungal **(B)** diversities of wrapping leaves based on the Chao1 and observe index. *Alstonia macrophylla* (Am), *Megaphrynium macrostachyum* (Mm), *Thaumatococcus daniellii* (Td), and *Theobroma* species (Ts).

**Figure 3:**
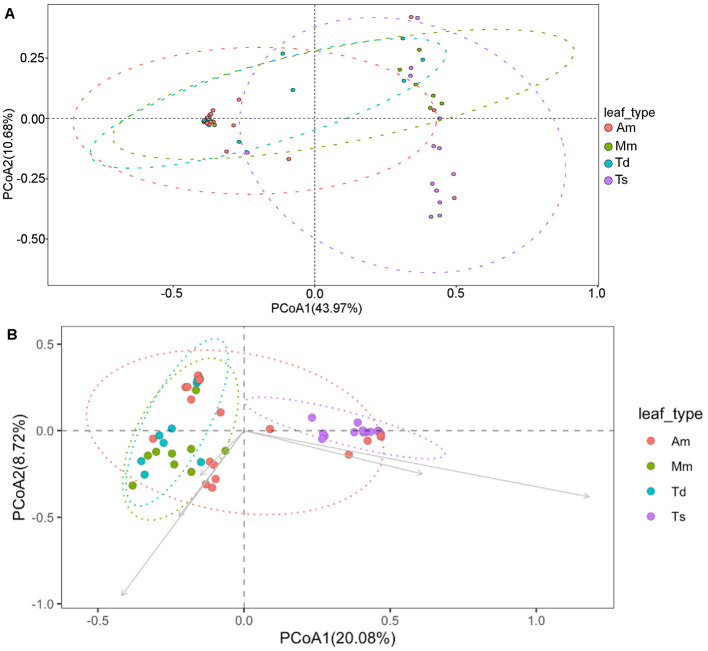
Principal coordinate analysis (PCoA) plot, showing the similarities in microbial beta diversity among wrapping leaves: **(A)** bacterial beta diversity; **(B)** fungal beta diversity using the Bray–Curtis dissimilarity. *Alstonia macrophylla* (Am), *Megaphrynium macrostachyum* (Mm), *Thaumatococcus daniellii* (Td), and *Theobroma species* (Ts).

**Figure 4:**
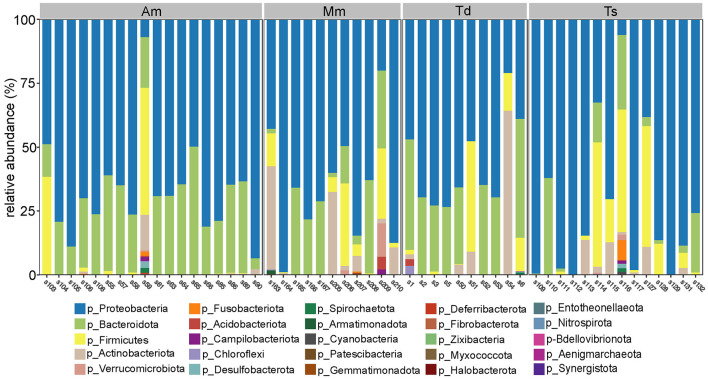
Relative abundance of bacteria from wrapping leaves at phylum level. *Alstonia macrophylla* (Am), *Megaphrynium macrostachyum* (Mm), *Thaumatococcus daniellii* (Td), and *Theobroma* species (Ts).

**Figure 5:**
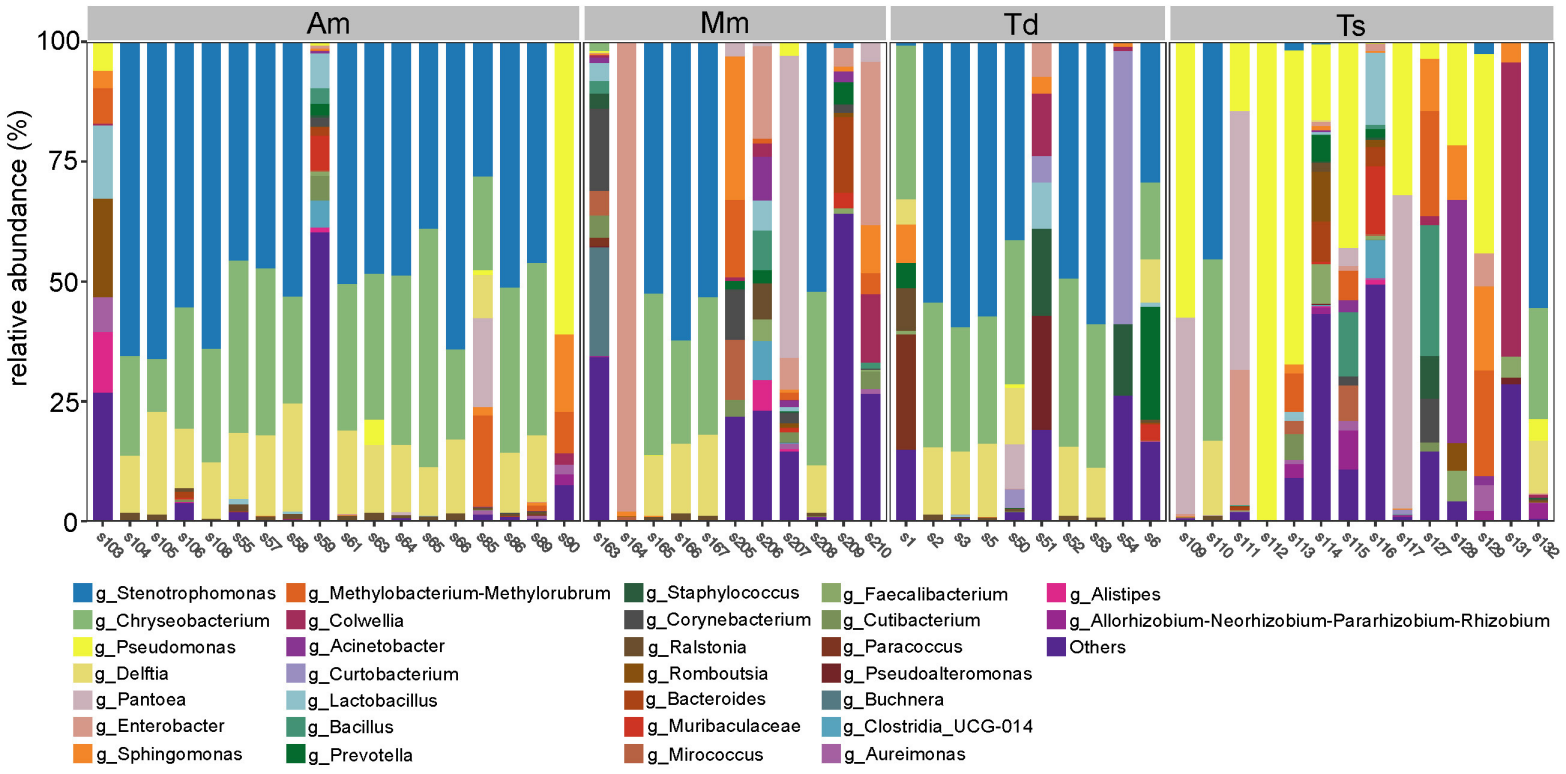
Relative abundance of bacteria from wrapping leaves at genus level. *Alstonia macrophylla* (Am), *Megaphrynium macrostachyum* (Mm), *Thaumatococcus daniellii* (Td), and *Theobroma* species (Ts).

**Figure 6:**
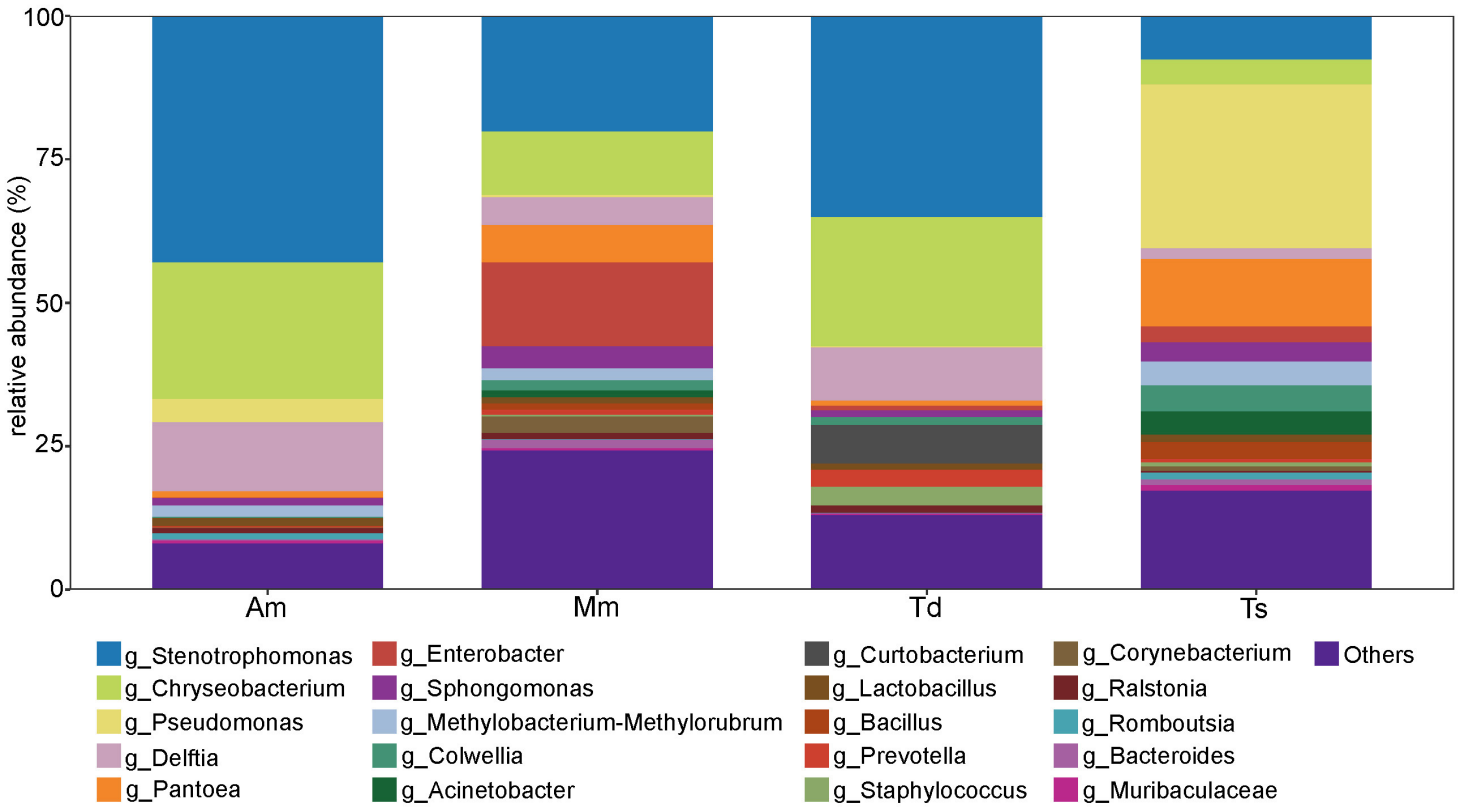
Top abundant 20 genera from wrapping leaves. *Alstonia macrophylla* (Am), *Megaphrynium macrostachyum* (Mm), *Thaumatococcus daniellii* (Td), and *Theobroma* species (Ts).

**Figure 7:**
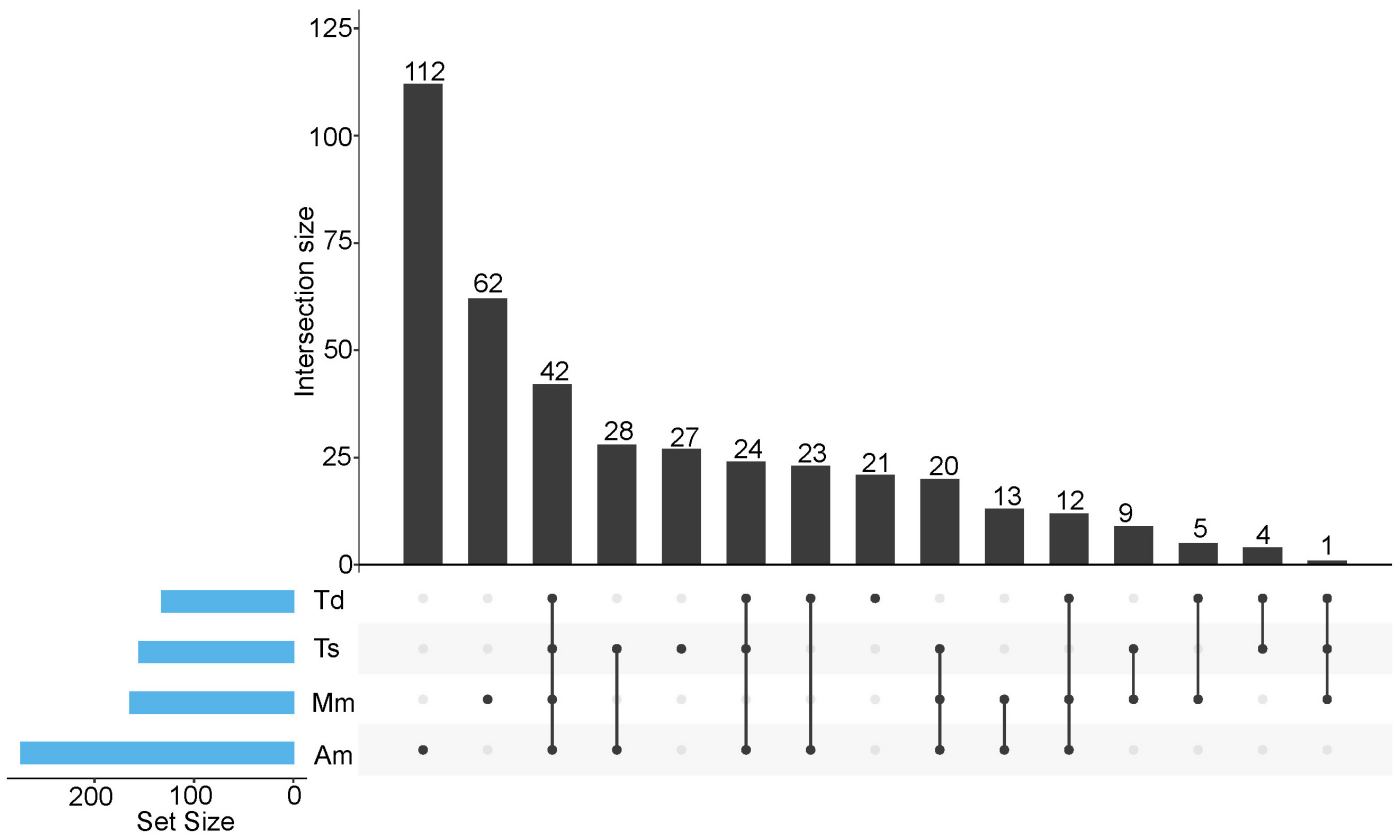
Upset plot showing shared and unique bacteria taxa in wrapping leaves. *Alstonia macrophylla* (Am), *Megaphrynium macrostachyum* (Mm), *Thaumatococcus daniellii* (Td), and *Theobroma* species (Ts).

**Figure 11:**
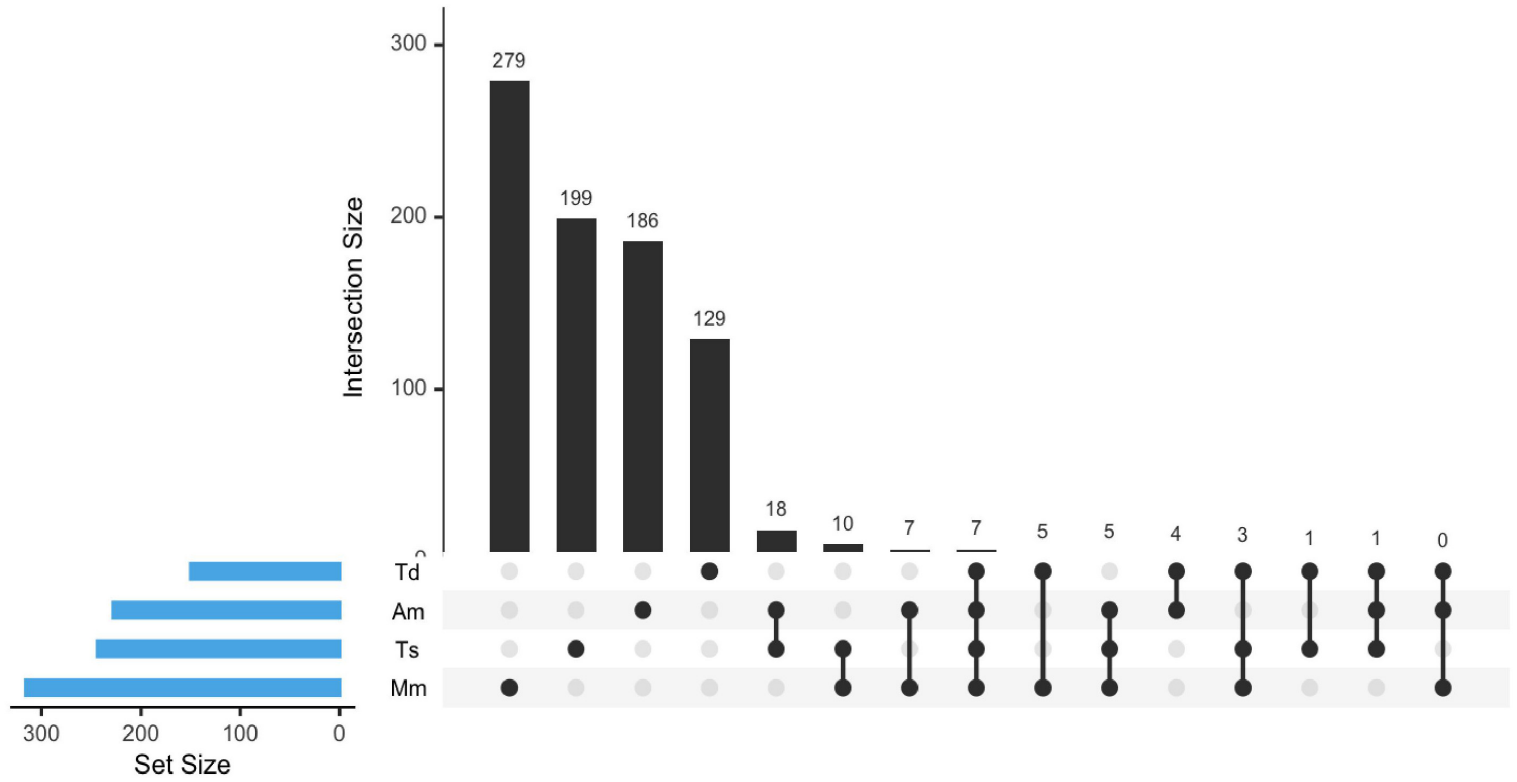
Upset plot showing shared and unique fungal taxa in wrapping leaves. *Alstonia macrophylla* (Am), *Megaphrynium macrostachyum* (Mm), *Thaumatococcus daniellii* (Td), and *Theobroma* species (Ts).

**Figure 12:**
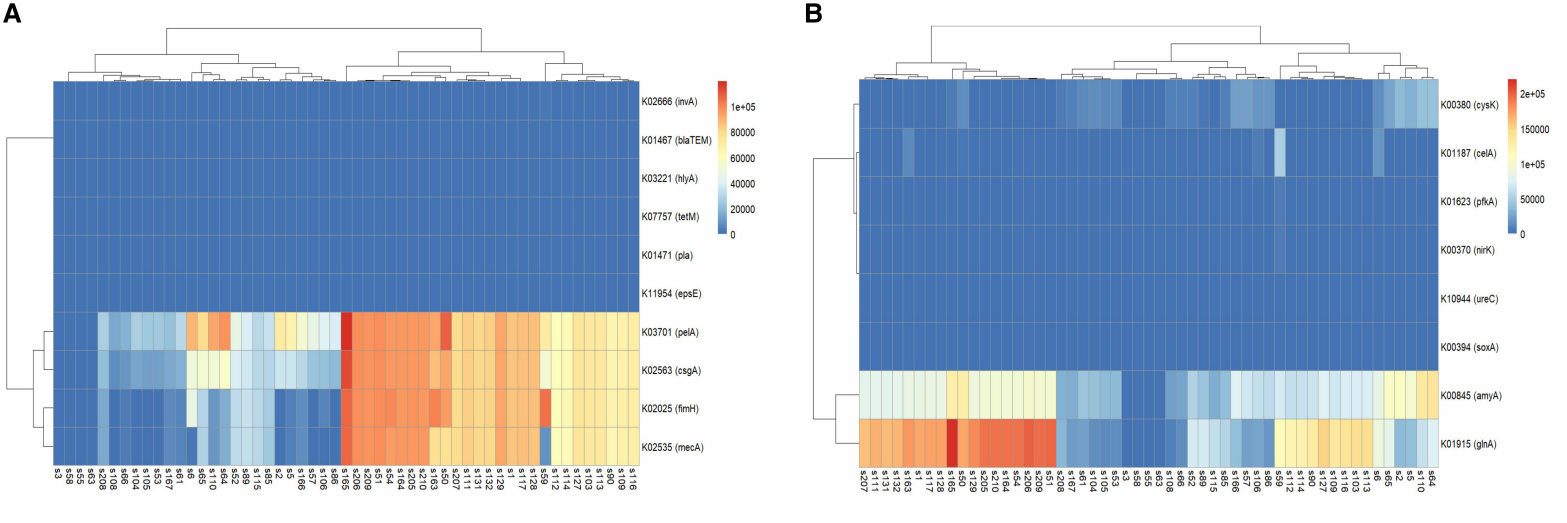
**(A)** Abundance of KEGG Orthology (KO) terms associated with nutrient metabolic pathways. **(B)** Abundance of KEGG Orthology (KO) terms associated with pathogenic pathways.

**Figure 13:**
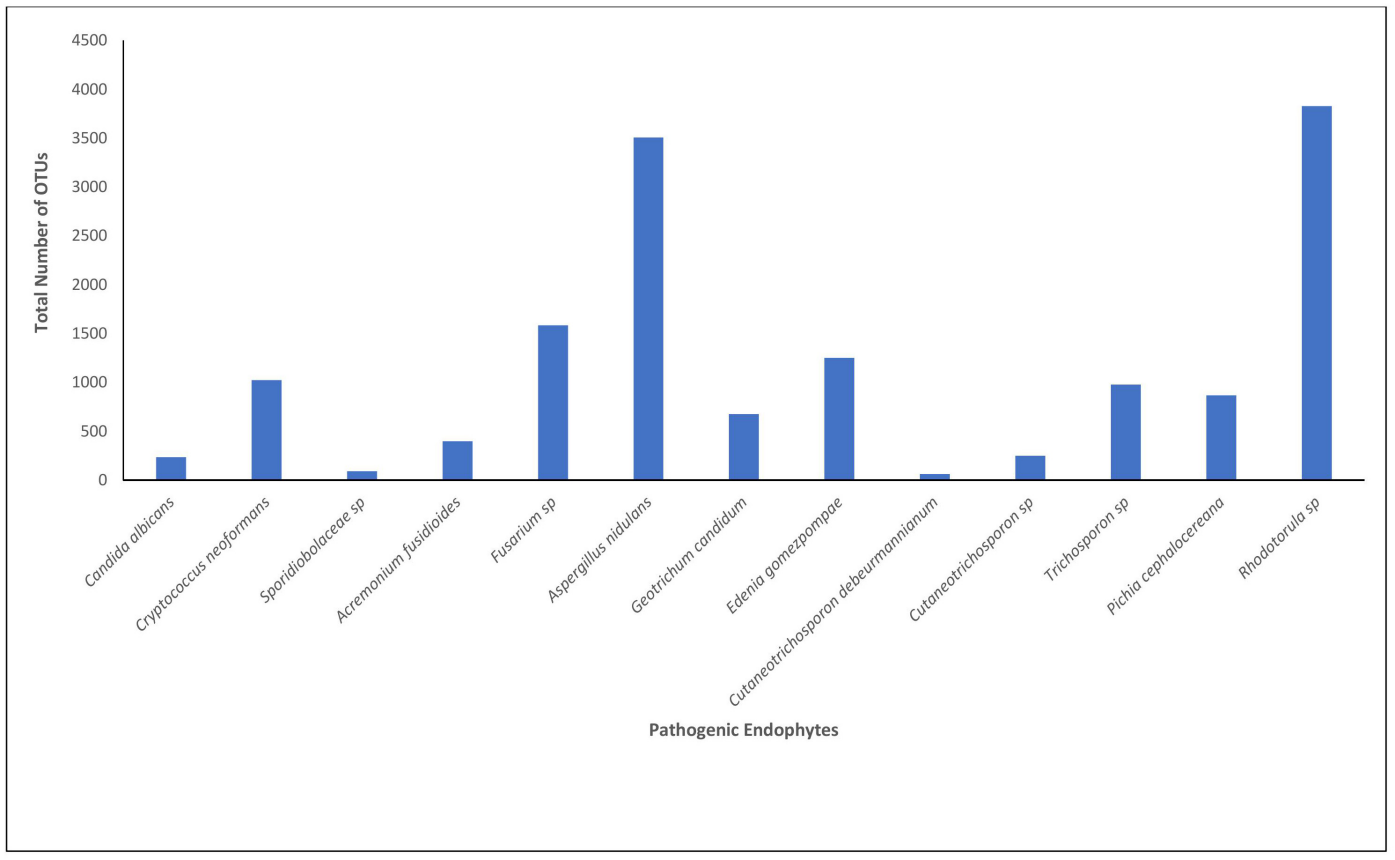
Number of OTUs of pathogenic fungi from wrapping leaves.

The original version of this article has been updated.

